# Self-diffusion and shear viscosity of pure 1-alkanol unary system: molecular dynamics simulation and review of experimental data†

**DOI:** 10.1039/d4ra03494e

**Published:** 2024-07-22

**Authors:** Adnan Jaradat, Rakan Al-Salman, Abdalla Obeidat

**Affiliations:** a Department of Physics, Jordan University of Science and Technology Irbid Jordan aobeidat@just.edu.jo

## Abstract

Self-diffusion coefficients and shear viscosity coefficients of pure 1-alkanol liquids from methanol to 1-hexanol were predicted using molecular dynamics (MD) simulations. These coefficients have been calculated using the Green–Kubo and Einstein methods at a range of temperatures of 200–330 K with increments of 10 K. Two force fields, TraPPE-UA and OPLS-AA were applied. The predicted results were compared to the experimental data, and the activation energies for self-diffusion and shear viscosity were calculated using the Arrhenius equation. The Stokes–Einstein equation was used to examine its capability in predicting the relationship between self-diffusion and shear viscosity, and the effective hydrodynamic radius was determined using both the experimental data and the results from MD simulations. The TraPPE-UA force field showed better results for the transport properties of methanol, while the OPLS-AA force field performed well for predicting shear viscosity but weakly for self-diffusion, particularly at low temperatures and for 1-alkanol with higher methylene numbers. Using the mean squared displacement method for self-diffusion was found to be more accurate than the Green–Kubo method, while the Green–Kubo method was slightly better for calculating shear viscosity. The Stokes–Einstein equation is valid for pure 1-alkanol liquids with temperature-dependent effective hydrodynamic radius.

## Abstract 2

Self-diffusion coefficients and shear viscosity coefficients of pure 1-alkanol liquids from methanol to 1-hexanol were predicted using molecular dynamics (MD) simulations.

## Graphical abstract

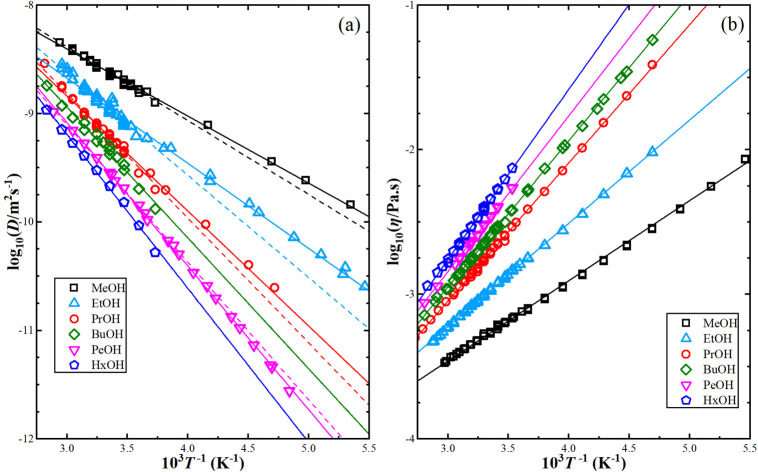

## 1. Introduction

Transport properties such as self-diffusion and shear viscosity are significant for understanding the behavior of pure and mixed liquids in various applications and these properties make 1-alkanol liquids (primary alcohol) useful as solvents, cleaning agents, medical and pharmaceutical industries, reactants and intermediates, and as biofuels and gasoline additives.^1,2^ In reviewing the experimental data on the self-diffusion^3–20^ and shear viscosity coefficients^21–47^ of pure 1-alkanols from methanol to 1-hexanol, we have found that there is a lack of data available at very low temperatures, with most data focusing on medium temperatures or around room temperature. There is also generally more experimental data available for shear viscosity coefficients than for self-diffusion coefficients, due to the relative ease and low cost of measuring viscosity.

The presence of the hydroxyl group (OH) in alcohols gives them polar properties and allows them to mix, making the study of their properties important. 1-Alkanols have a hydroxyl group on one side of the molecule and a methyl group (CH_3_) on the other side, and the number of methylene groups (CH_2_) between functional groups in a molecule which plays a crucial role in determining several physical properties, including melting and boiling points,^48^ solubility,^49^ density,^50^ viscosity,^51^ molecular flexibility,^52^ and crystallinity.^53^ For instance, 1-hexanol, which has five methylene groups, exhibits different properties compared to methanol, which has no methylene groups. These differences in the molecular structure of 1-alkanols significantly affect their properties. As the number of methylene groups increases, the carbon chain length also increases, leading to changes in molecular size, shape, and interactions. Therefore, the number of methylene groups is a key factor in determining the physical properties of these chemical families, influencing how the molecules behave in various environments and applications.

On the other hand, molecular dynamics (MD) simulations became widely used for calculating various physical properties of liquids. The challenges associated with this method are the need for long periods and large amounts of storage capacity for data, as MD simulation is based on the analysis of a massive amount of statistical data, and the accuracy of the measurements is related to the number of actual MD steps. Recently, advances in computing capabilities, particularly the graphics processing units (GPUs), have helped to alleviate these challenges and have made MD simulations a more reliable and accurate method with a reduced margin of error.^54^

MD early studies focused on understanding the hydrogen-bonding network in 1-alkanols, such as methanol, ethanol, and higher 1-alkanols, and the simulations revealed that hydrogen bonds significantly influence the structure and dynamics of these molecules.^55^ Several studies have utilized MD simulations to predict the viscosity of 1-alkanols and have demonstrated good agreement with experimental data. For example, Guevara-Carrion *et al.*^56^ employed equilibrium molecular dynamics (EMD) simulations to calculate the shear viscosity of methanol and ethanol, achieving deviations of approximately 8% from experimental values. This study highlighted the dominance of potential energy contributions to the overall viscosity values for these alcohols. In another study by Fan *et al.*^57^ using MD simulation methods, the researchers successfully estimated the thermal transport properties of alcohols, including thermal conductivity and viscosity. They demonstrated that these methods could be an effective and accurate alternative to experimental methods in situations where experiments are not feasible. In a study by Zhang *et al.*,^58^ an innovative method was introduced to improve the accuracy of viscosity calculations for liquids using MD simulations. Instead of relying on a single long trajectory, they divided the simulation into several short independent trajectories. This method was applied to liquid ethanol over a range of temperatures, and the results showed that the method is effective in producing reliable and consistent viscosity values.

The self-diffusion coefficients of 1-alkanols have also been extensively studied using MD simulations. Li *et al.*^59^ investigated the self-diffusion coefficients of methanol, ethanol, 1-propanol, 2-propanol, and 1-pentanol over a range of temperatures and pressures. Their findings showed that the calculated self-diffusion coefficients generally conformed to experimental values, with temperature having a more significant impact on the coefficients than pressure. Feng *et al.*^60^ used molecular dynamics simulations to investigate the self-diffusion coefficients and local structures of simple alkanols (methanol, ethanol, and 2-propanol) across a range of temperatures and pressures, finding that temperature has a more significant impact than pressure on both diffusion and structural properties, including hydrogen bonding and coordination numbers. Another study using MD simulations on 13 aliphatic alcohols done by Kulschewski and Pleiss^61^ evaluated properties such as density and self-diffusion coefficients across temperatures from 288 to 338 K. The OPLS all-atom force field accurately reproduced experimental densities with deviations less than 4%. Modifying hydroxyl group charges reduced self-diffusion coefficient deviations from 55% to less than 19%.

In our previous work,^62–65^ we studied the properties of pure 1-alkanol and its mixtures with water using various models, most notably the OPLS-AA^66^ and TraPPE-UA^67^ models. These models demonstrated good efficiency in predicting different physical properties when compared to experimental values. This has led us to use these two models in our current study. The accuracy of MD simulations depends on the force fields used to describe intermolecular interactions, with commonly used force fields for 1-alkanols including OPLS and CHARMM.^66^ Additionally, MD simulations have been employed to compute the viscosity of 1-alkanols and compare them with experimental data, helping to understand how viscosity increases with chain length.^68^

Overall, our study aims to provide an understanding of the influence of methylene groups on the transport properties of pure 1-alkanol liquids and improve the accuracy of MD simulations for predicting these properties, and to compare the usefulness of the two force fields, namely OPLS-AA^66^ and TraPPE-UA,^67^ and the efficacy of Green–Kubo (GK)^69,70^*versus* mean-squared displacement (MSD) methods. Also, we aim to investigate the effect of increasing the number of methylene groups (*i.e.*, carbon atoms) in pure 1-alkanol chains on the self-diffusion and shear viscosity within a temperature range of 200–330 K. We will compare experimental data from various sources with the simulation results obtained using MD. On the other hand, we aim to study the relationship between viscosity and self-diffusion, and test the validity of the Stokes–Einstein^71^ equation for 1-alkanol systems by using the available data from the literature, and our MD results. Such knowledge is important for optimizing the use of 1-alkanols in a variety of applications and for developing new materials with enhanced transport properties. Calculating the coefficient of self-diffusion using MD simulation is generally easier and less computationally expensive than calculating viscosity.^72^

## 2. Review of experimental data

Shear viscosity is the amount of a liquid's resistance to flow under applied shear stress and is related to the frictional forces within the liquid, on the other hand, self-diffusion is the movement of particles within a substance due to their thermal motion and is a measure of the random motion of particles within a liquid. Self-diffusion and shear viscosity coefficients depend on temperature, generally, this dependence can be expressed by well known Arrhenius equation^73^ as follow.1a
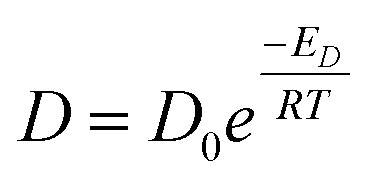
1b
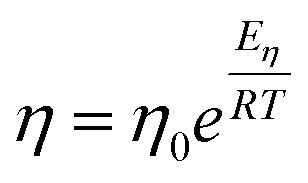
where *D*_0_ and *η*_0_ are the pre-exponential factors, which represents the maximal self-diffusion coefficient at infinite temperature, and the viscosity value that the liquid would approach if the temperature were to become extremely high (at infinite temperature) respectively, *T* is the temperature, *R* is the gas constant (8.31446 J K^−1^ mol^−1^), where *E*_*D*_ and *E*_*η*_ are the activation energies of diffusivity and viscosity, respectively. Previous equations can be rewritten as a linear form equation as follows.2a
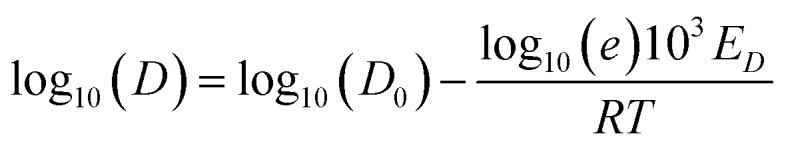
2b
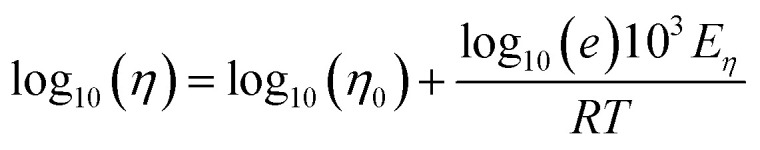
where in this form, *E*_*D*_ and *E*_*η*_ are given in kJ mol^−1^.

It is important to compare the results of MD simulations with experiments to estimate the accuracy of the computer models in calculating different physical and chemical properties. Additionally, it is necessary to compare multiple experimental studies to assess the reliability of these studies and the consistency between them. The differences between experimental results may be due to variations in the measurement method, data processing, and measurement errors. In this study, the activation energies for shear viscosity and self-diffusion were calculated for each experimental study by fitting the data to the Arrhenius equation using linear least-squares fitting. The fitting results are shown in Table 1, along with margins of error for the line slopes to indicate how well the data follow the Arrhenius equation. The activation energies for shear viscosity from different studies are consistent, but there are some outlier points for self-diffusion, particularly at low temperatures. When these outlier points are plotted alone, they form a straight line (see ESI Fig. S1†), which raises questions about the accuracy of self-diffusion measurements at low temperatures, especially since self-diffusion values are small at low temperatures. To improve the correlation of the total data, secondary calculations were performed by excluding the self-diffusion values at low temperatures below 260 K and comparing them with the results obtained without excluding these values. We did not encounter similar issues when calculating the activation energy for shear viscosity, the agreement among the data for this property from all sources used in this work was within acceptable limits, unlike the self-diffusion data. This makes sense considering the different measurement methods used to calculate the two properties.

**Table 1:** Self-diffusion and shear viscosity experiment data sources used in this study are listed, along with the number of points (*n*), temperature range, and our work calculated activation energies of self-diffusion (*E*_*D*_) and shear viscosity (*E*_*η*_) from Arrhenius plots. (Note: in this table and our calculations, we ignore the data linked with temperatures above 360 K and under 180 K)

Self-diffusion					Shear viscosity				
*E*_*D*_ (kJ mol^−1^)	T-rang (K)	*n*	Year	Ref.	*E*_*η*_ (kJ mol^−1^)	T-rang (K)	*n*	Year	Ref.
**Methanol**									
11.72 ± 0.07	288.15–308.15	3	1952	3	10.51 ± 0.22	276.92–336.41	13	1894	21
14.85 ± 0.81	288.15–313.15	3	1958	4	10.81 ± 0.74	176.15–303.15	15	1967	25
13.85 ± 0.18	268.15–328.15	7	1961	5	10.83 ± 0.62	183.15–283.15	11	1971	27
13.26 ± 0.13	298.15–328.15	4	1977	6	10.37 ± 0.21	288.15–328.15	5	1983	30
12.72 ± 0.18	278.2–328.2	9	1985	7	10.63	303.15–308.15	2	2000	37
12.01 ± 0.05	187–292	5	1990	8	10.44 ± 0.22	303.15–323.15	5	2004	41
11.00 ± 0.34	288.15–308.15	3	1996	9					
13.33	288 340	2	1998	10					
11.69 ± 0.58	273.1–298	3	2003	11					

**Ethanol**									
18.92 ± 0.54	288.15–318.15	4	1952	12	14.21 ± 0.33	280.31–346.72	12	1894	21
19.43 ± 0.08	288.15–308.15	3	1952	3	13.57 ± 0.51	213.15–293.15	9	1926	22
19.10 ± 0.34	279.95–338.15	7	1961	5	14.09 ± 0.30	273.15–348.15	16	1970	26
19.38	298.15,318.15	2	1965	13	13.88 ± 0.32	288.15–328.15	5	1983	30
15.67 ± 0.22	188.55–333.15	9	1970	14	14.15	303.15–308.15	2	2000	37
19.07 ± 0.50	298.15–338.15	5	1977	6	14.47 ± 0.74	303.15–323.15	5	2004	41
16.82 ± 0.46	287.8–317.8	5	1988	15	13.76 ± 0.68	283.15–313.15	4	2013	44
15.12 ± 0.19	183–333	8	1990	8					
17.02 ± 1.34	288.15–308.15	3	1996	9					
17.14 ± 0.65	288.15–303.15	4	2000	16					
15.67 ± 0.08	273.1–298	3	2003	11					

**1-Propanol**									
17.82 ± 0.07	288.15–318.15	4	1952	3	18.37 ± 0.15	280.46–368.74	14	1894	21
22.84 ± 0.31	298.15–338.15	5	1977	6	18.40 ± 0.48	213.15–293.15	9	1926	22
23.99 ± 0.51	287.8–317.8	5	1988	15	18.48	288.15–303.15	2	1934	23
22.28 ± 0.37	212–293	6	1993	17	16.83 ± 2.09	288.15–328.15	5	1983	30
19.49 ± 0.18	288.15–303.15	4	2000	16	18.82 ± 0.24	298.15–313.15	4	1995	32
19.82 ± 0.42	268.15–353.15	9	2010	18	17.51	293.15–298.15	2	1999	36
					17.15 ± 0.15	303.15–313.15	3	2000	37
					19.31	303.15–313.15	2	2003	40
					16.94 ± 0.66	303.15–323.15	5	2004	41

**1-Butanol**									
19.29 ± 0.02	298.15–318.15	3	1952	3	19.33 ± 0.10	273.42–356.28	9	1894	21
22.84 ± 0.74	288.15–303.15	4	2000	16	20.02 ± 0.24	303.15–333.15	4	1973	28
23.56 ± 0.40	268.15–353.15	9	2010	18	18.92 ± 0.92	223.15–263.15	5	1975	29
					18.41 ± 1.28	288.15–328.15	5	1983	30
					19.32 ± 0.54	203.15–384.35	10	1991	31
					20.01 ± 0.17	298.15–313.15	4	1995	32
					19.43	298.15–313.15	2	1996	33
					19.18 ± 0.01	293.15–313.15	3	1998	34
					19.34	293.15–298.15	2	1999	36
					19.72 ± 0.34	303.15–323.15	3	2000	38
					19.07 ± 0.68	303.15–313.15	3	2000	37
					18.03	303.15–313.15	2	2003	40
					18.83 ± 0.32	303.15–323.15	5	2004	41
					19.27 ± 1.03	283.15–313.15	5	2013	44

**1-Pentanol**									
24.12 ± 0.09	206.6–346.5	17	1995	19	20.30 ± 0.34	298.05–356.25	5	1963	24
25.29 ± 0.20	278.15–328.15	6	2000	20	21.37	293.15–298.15	2	1999	36
					22.33 ± 3.79	303.15–323.15	3	2000	38
					21.57 ± 0.51	303.15–313.15	3	2000	37
					20.17 ± 2.69	303.15–323.15	5	2004	41
					21.40 ± 0.38	283.15–313.15	5	2013	44
					21.06 ± 0.15	283.15–313.15	5	2013	43
					21.01 ± 0.21	293.15–323.15	4	2020	46

**1-Hexanol**									
27.17 ± 0.68	268.15–353.15	9	2010	18	22.02 ± 0.78	303.15–323.15	3	2000	38
					22.43 ± 2.91	303.15–353.15	3	2002	39
					22.27 ± 0.29	303.15–323.15	5	2004	41
					22.83 ± 1.54	283.15–343.15	7	2008	42
					22.71 ± 0.16	293.15–323.15	7	2018	45
					22.40 ± 0.27	288.15–338.15	11	2020	47
					22.74 ± 0.25	293.15–323.15	4	2020	46

**Fig. 1:**
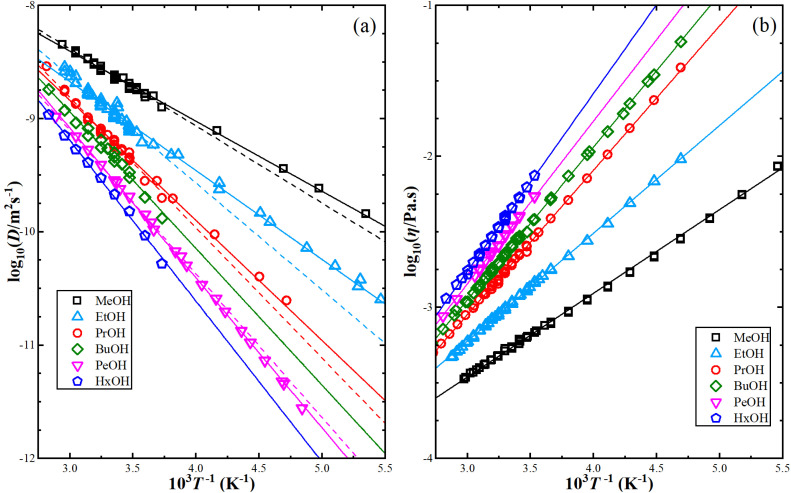
Arrhenius linear form plot of the self-diffusion (a) and shear viscosity (b) for methanol (MeOH), ethanol (EtOH), 1-propanol (PrOH), 1-butanol (BuOH), 1-pentanol (PeOH), and 1-hexanol (HxOH). Solid lines denote the linear fitting using all points, and dash lines for linear fitting using points within temperatures above 260 K.

Fig. 1 illustrates the dependence of experimental shear viscosity and self-diffusion on the temperature in the form of the linear Arrhenius equation for pure 1-alkanol liquids ranging from methanol to 1-hexanol (the exponential forms are shown in ESI Fig. S2†). The parameters in the Arrhenius equation can be extracted by calculating the slope and intersection of the straight line resulting from the linear fitting process of eqn (2a) and (2b). The figure demonstrates the high efficiency of the Arrhenius equation for all types of 1-alkanols at all temperatures for shear viscosity data and shows an increase in the activation energy of shear viscosity (slope of each curve) with an increase in methylene groups (CH_2_) number. However, the results of the Arrhenius equation fitting for self-diffusion show lower quality due to the divergence of experimental readings from one source to another. The activation energy of self-diffusion also appears to differ when ignoring data points associated with temperatures less than 260 K. This is particularly noticeable in the case of methanol, ethanol, and 1-propanol but is less apparent in the case of 1-pentanol. No readings at lower than 260 K for 1-butanol and 1-hexanol were found in the literature for comparison. These outcomes raise questions about the efficiency of techniques used to measure self-diffusion at extremely low temperatures where the conditions of the experiment are more difficult, and the values of self-diffusion are negligible compared to values at higher temperatures. The fitting parameters of the Arrhenius equation for both shear viscosity and self-diffusion (both ignoring and not ignoring self-diffusion data below 260 K) are reported in Table 2.

**Table 2:** Fitting parameters of the experimental data from the Arrhenius and the Stoke–Einstein equations, this data will be used as correlated experimental equations for comparison with MD results in the later parts

	*D*_0_ (10^−6^ m^2^ s^−1^) (all *T*)	*D*_0_ (10^−6^ m^2^ s^−1^) (*T* > 260 K)	*η*_0_ (10^−6^ Pa s^−1^)	*E*_*D*_ (kJ mol^−1^) (all T)	*E*_*D*_ (kJ mol^−1^) (*T* > 260 K)	*E*_*η*_ (kJ mol^−1^)	*α* (nm) average (*T*: 280–320 K)
Methanol	0.28 ± 0.02	0.46 ± 0.05	7.54 ± 0.22	11.85 ± 0.125	13.05 ± 0.30	10.61 ± 0.06	0.170 ± 0.001
Ethanol	0.49 ± 0.04	1.60 ± 0.34	4.30 ± 0.09	15.05 ± 0.175	18.07 ± 0.43	13.68 ± 0.05	0.188 ± 0.002
1-Propanol	2.21 ± 0.29	4.33 ± 0.84	1.21 ± 0.04	20.30 ± 0.317	22.02 ± 0.75	18.31 ± 0.08	0.188 ± 0.002
1-Butanol	4.79 ± 1.65	4.79 ± 1.65	1.03 ± 0.01	23.10 ± 0.851	23.10 ± 1.24	19.38 ± 0.05	0.194 ± 0.004
1-Pentanol	7.73 ± 0.53	5.02 ± 0.84	0.84 ± 0.01	25.34 ± 0.146	24.28 ± 0.46	20.64 ± 0.10	0.223 ± 0.003
1-Hexanol	11.65 ± 3.15	11.65 ± 3.15	0.51 ± 0.01	27.18 ± 0.678	27.18 ± 0.52	22.55 ± 0.14	0.226 ± 0.001

The shear viscosity can be related to self-diffusion by the famous Stoke–Einstein relation^71^ expressed as follow.3
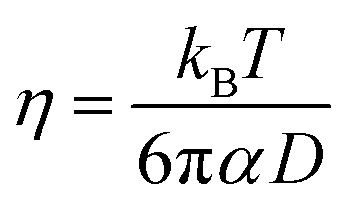
where *k*_B_ is the Boltzmann constant, *T* is the absolute temperature, and *α* is the effective hydrodynamic radius.

**Fig. 2:**
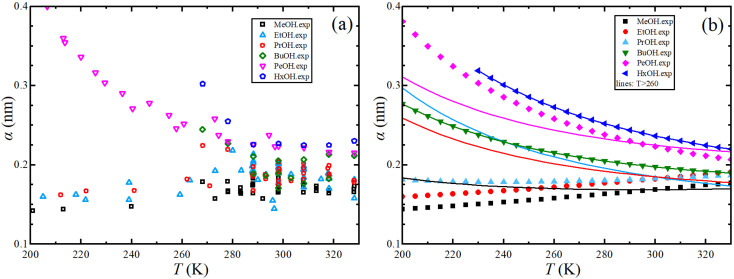
(a) Experimental effective hydrodynamic radius temperature dependence of 1-alkanol from methanol to 1-hexanol. (b) Same but by using the Arrhenius equation fitting parameter for self-diffusion and shear viscosity listed in Table 2 by using all data of self-diffusion (symbols) and the data related to the temperatures above 260 K (solid lines).

The Stokes–Einstein equation describes the self-diffusion coefficient of an isolated spherical particle undergoing Brownian motion in a continuum fluid with shear viscosity, and with the stick boundary condition at the particle surface. This equation is valid under the assumption that the effective hydrodynamic radius is constant as a function of temperature, which leads to a linear relationship between self-diffusion and temperature divided by viscosity. However, many studies have shown that the effective hydrodynamic radius for liquids is not constant with temperature, especially at extremely low temperatures.^74–76^ In this work, to plot the relationship between effective hydrodynamic radius and temperature, we need the values of viscosity and self-diffusion coefficients at each temperature. To obtain the corresponding values for each experimental value of self-diffusion at a certain temperature, the linear fitting data in Table 2 were used to calculate the viscosity at the same temperature of self-diffusion that was originally available in the literature. Fig. 2a shows the temperature dependence of the effective hydrodynamic radius (*α*) for 1-alkanols from methanol to 1-hexanol, indicating that the Stokes–Einstein equation does not hold strictly since *α* is not constant but varies with temperature. This temperature dependence is more evident at low temperatures, assuming the experimental data at these temperatures is accurate. Fig. 2b shows effective hydrodynamic radius temperature dependence by the Arrhenius equation fitting parameter for self-diffusion and shear viscosity listed in Table 2 by using all data of self-diffusion and the data related to the temperatures above 260 K. The relationship between shear viscosity and temperature divided by self-diffusion for each 1-alkanol system is shown in ESI Fig. S3.† The average effective hydrodynamic radius at temperatures range of 280–320 K is calculated and reported in Table 2.

## 3. Molecular dynamics simulations methodology

### 3.1. Potential models

In this study, we chose the best two potential models based on their success in our previous works;^62–64^ the Optimized Potentials for Liquid Simulations all-atoms (OPLSAA)^66^ and Transferable Potentials for Phase Equilibria unite-atom (TraPPE-UA)^67^ force fields. The number of interaction sites in a TraPPE-UA force field is designed to be as small as possible without losing excessive accuracy. For 1-alkanol single interaction, sites represent a carbon atom together with all its bonded hydrogen atoms (*i.e.*, CH_3_, CH_2_). However, hydroxyl (OH) atoms are treated as specific interaction sites to keep the polarity on the whole molecule. This is a big advance for the TraPPE-UA force field by reducing the computational cost spatially with long molecule chains, for example, a 1-hexanol molecule has twenty-one interaction sites in the OPLS-AA force field and eight interaction sites TraPPE-UA force field, and this will increase the performance of the TraPPE-UA force field by approximately threefold under the same computational environment. Single-molecule snapshots for all 1-alkanol used in this work are shown in the ESI Fig. S4.† The intermolecular potential between atoms *i* and *j* is the sum of Lennard–Jones (LJ)^77^ and Coulomb potentials:4
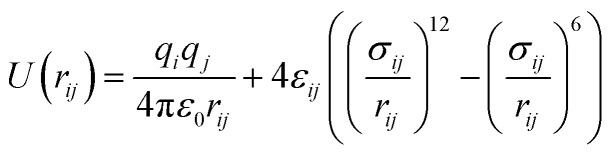
where *ε*_*ij*_ and *σ*_*ij*_ are Lennard–Jones parameters which represent the van der Waals radius and the depth of the potential, respectively. The Lorentz–Berthelot rules are applied for the different kinds of atoms interaction (*σ*_*ij*_ = (*σ*_*i*_ + *σ*_*j*_)/2 and 
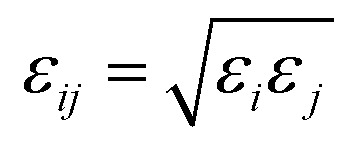
). The Lennard–Jones parameters and the geometries of the TraPPE-UA and OPLS-AA force fields are listed in ESI Table S1.†

### 3.2. Transport properties

To obtain shear viscosity and self-diffusion coefficients by using the Green–Kubo^69,70^ equation in equilibrium MD simulation, pressure autocorrelation functions (PACF) and velocity autocorrelation functions (VACF) must be calculated in the first place. The formulas for computing a transport property *via* equilibrium MD simulations can be expressed as:5a
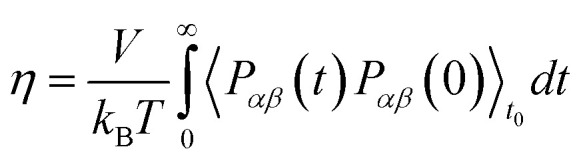
5b
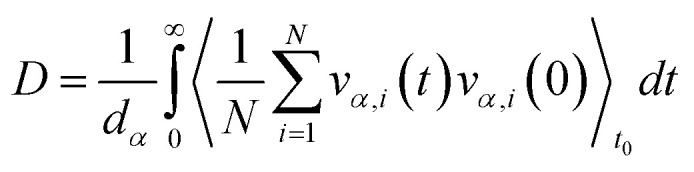
where *V* is the volume of the system, *t*, and *t*_0_ are the time and the time origin respectively. *P*_*αβ*_ is the element of the off-diagonal pressure tensor. *α* and *β* are *x*, *y*, or *z* the cartesian coordinates of the whole system with the role of *α* ≠ *β*, *d*_*α*_ is the dimensionality (1, 2, or 3), *υ*_*α*,*i*_ is the translational velocity of the atom “*i*” (or molecule center of mass) in *α* cartesian direction, *N* is the number of atoms or molecules in the system, the alternative method to obtain shear viscosity and self-diffusion coefficients from an equilibrium simulation is by using an Einstein relation, the mean square displacement and the mean square of the off-diagonal pressure tensor are required as:6a
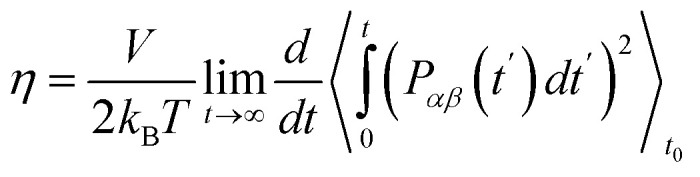
6b
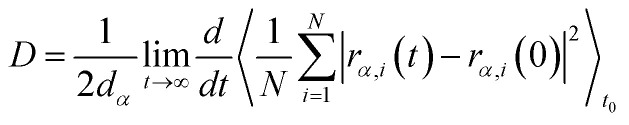
where *r*_*α*,*i*_ is the position of the atom *i* (or molecule center of mass) in *α* cartesian direction. Eqn (6a) and (6b) can be derived from eqn (5a) and (5b) respectively, therefore, the two methods theoretically are equivalent,^78^ but the strategies in the MD simulations may be different from one method to another based on the amount and type of the statistical trajectories used to average. The strategy used in the MD simulations differs in the process of calculating both the shear viscosity and the self-diffusion. In contrast to the self-diffusion, which is an independent property of each particle present in the system, the shear viscosity is a collective property of the entire system, and therefore, to calculate the viscosity we need to run multiple attempts to obtain good statistics,^58^ and the more attempts led to better results, but this is associated with a greater computing time cost. Also, the two methods used in calculating the viscosity slowly converge to a stable point, and usually, we need a time greater than 1 ns for each attempt to give better results. In the self-diffusion calculations, the VACF curve goes to zero quickly up to several picoseconds, despite that, increasing the simulation time will increase the statistical values used in this process, and conducting several independent attempts increases the quality of the results, which is somewhat different from using Einstein method, as *t* decreases the VACF converges faster than the mean square displacement, so we need a time of up to several ns to get better results.

### 3.3. Simulation details

All simulations in this work were done using GROMACS 2018.8 package.^54^ For self-diffusion coefficient calculation, 1000 molecules of ethanol, methanol, 1-propanol, 1-butanol, 1-pentanol, and 1-hexanol are confined randomly as pure systems cubic boxes of (4.1 nm)^3^, (4.6 nm)^3^, (5.0 nm)^3^, (5.4 nm)^3^, (5.6 nm)^3^ and (5.9 nm)^3^ sizes respectively. As the number of molecules in MD simulation does not affect the shear viscosity value;^58,72^ the number of molecules reduced to 500 molecules in computing shear viscosity to reduce computational time cost as the calculations need many repeating times to get more accurate values of shear viscosity. For each system, energy minimization (EM) is used to ensure that the system is relaxed without any steric crashes or unsuitable geometry that disagree with the chosen potential model. In this work, the steepest descent algorithm^79^ was used with maximum steps of 200 000 energy minimization and with a 2 × 10^−5^ kJ mol^−1^ energy step.

In this work in order to calculate both viscosity and self-diffusion using MD simulation, the goal is to obtain a large amount of statistical data for both the position and the velocity of each atom in each characteristic time to calculate the self-diffusion and the amount of pressure tensor to calculate the viscosity, these data must be collected after making sure that the system is in thermal equilibrium and this can be justified by performing the equilibrium process within two steps, the first step is to change the size of the initial box by applying pressure to obtain density system that simulate the real system, this constant-pressure step (NPT) done by using the Parrinello–Rahman approach,^80,81^ and the second step is to move the contents of the box under a constant temperature and fixed size, this canonical ensemble equilibrium (NVT) done by using the Nosé–Hoover^82,83^ temperature thermostat, the final box sizes and bulk densities for all systems in this work are listed in the ESI Table S2.† The uncertainty values listed in this table were derived by calculating the standard deviation of the box dimensions' fluctuations over the last 50 ps of the NPT equilibration process. Densities were calculated based on the principle that density equals mass divided by volume. For comparison, experimental density values from ref. 84 were also added.

2 ns NPT followed by 2 ns NVT applied to equilibrate all our systems, all productions run after the equilibrium done as NVT ensembles. For both equilibrium and data collections, 1 bar reference pressure was applied, a 2 fs time step was applied, edge effects of our small finite box were minimized by applying periodic boundary conditions, which prevent the artifacts associated with finite system boundaries and more accurately represent an infinite bulk phase. And leap-frog algorithm integrator^85^ was used for the integral equations of the of motion, LINCS algorithm^86^ was used to correct bonds lengths after every update during simulations. A spherical cut-off radius of 1.3 nm has been used for the Leonard Jones short-range interaction and the PME Coulomb long-range interaction for the OPLS-AA model, and 1.4 nm for TraPPE-UA where this value is used as recommended by the inventors.^66,67^

Self-diffusion coefficients were calculated using the Green–Kubo method after collecting the data for 100 ps and saving the frames at every time step, this step was repeated 8 times and the average of VACF was taken from this trial with its stander deviation, also Self-diffusion coefficients were calculated using the Einstein relation by saving atoms coordinates of 6 ns run every 100 steps and then the time dependence of the mean square displacements was calculating. Sixteen repeating of 1*n* simulation times were done for shear viscosity calculations for both Green–Kubo and Einstein, the pressure tensors were calculated for every step, the time average for all sixteen integrals was calculated and the standard deviation of the average time integral was calculated as an uncertainty of the shear viscosity value. MD simulation diagram is provided in the ESI (Fig. S5†).

## 4. Results and discussions

In this section, four main data sets from MD simulation were studied for each 1-alkanol system from methanol to 1-hexanol namely, TraPPE-UA by the GK method, TraPPE-UA by MSD method, OPLS-AA by GK method, and OPLS-AA by MSD method, where GK denoted to Green–Kubo relation method and MSD denoted to Einstein relation method. A temperature ranging between 200–330 K with a 10 K step was used in calculating shear viscosity and self-diffusion. The technique of calculating the self-diffusion coefficient based on the calculation of the slope of the main square displacements when we use the Einstein method and the integral of the velocity autocorrelation when we use the Green–Kubo method, some examples of MSD and VACF profiles are listed in the ESI Fig. S6.† For accurate calculation of self-diffusion by the GK method, 8 repetitions were done and all results of this repetition are shown in the ESI Fig. S7† on the other hand, off-diagonal pressure tensor needed for share viscosity calculation, unlike VACF integral in the self-diffusion calculation, share viscosity integrals by both methods are converged slowly, the average time dependence integral values of shear viscosity examples are shown in the ESI Fig. S8,† and examples of 16 independent trials for time dependence integral shear viscosity values for single system are shown on the ESI Fig. S9.† And a comparison of MD shear viscosity results between this study and some results from other studies presented in the introduction is shown in Table S3 in the ESI.†

**Fig. 3:**
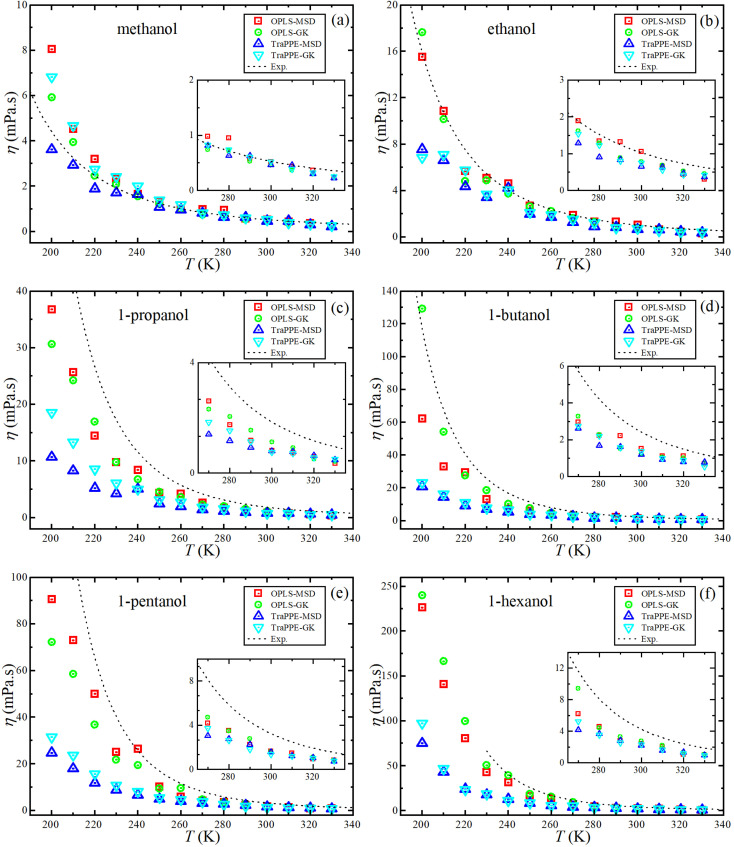
Temperature dependence of the predicted shear viscosity from MD simulations for pure 1-alkanol (a–f) liquids by Green–Kubo (GK) and Einstein (MSD) methods for OPLS-AA and TraPPE-UA force fields in comparison to a correlation of experimental data (dash lines).

Fig. 3 shows the dependence of shear viscosity on temperature from MD simulations for 1-alkanol systems, ranging from methanol to 1-hexanol, within a temperature range of 200–330 K. The simulations were performed using the Green–Kubo (GK) and Einstein (MSD) methods for OPLS-AA and TraPPE-UA force fields, and the results are compared to correlation equations of the experimental data. In general, the figure shows that OPLS-AA is much better than TraPPE-UA and the difference becomes more clear when the number of carbon atoms of alcohol increases, and this leads us to judge that TraPPE-UA cannot predict high shear viscosity values at relatively very low temperatures. Also, both force fields show a decrement in the shear viscosity values compared with the experimental values at all temperatures except for methanol, where there is an increase in the shear viscosity at low temperature of the force field of OPLS-AA by both methods and the force field of TraPPE-UA by GK method, and a small decrease in the values of the TraPPE-UA using the MSD method.

In general, the figure shows that OPLS-AA is much better than TraPPE-UA, and the difference becomes more evident as the number of carbon atoms in the alcohol increases. This suggests that TraPPE-UA cannot accurately predict high shear viscosity values at relatively low temperatures. Both force fields show a decrease in shear viscosity values compared to experimental values at all temperatures, except for methanol, where there is an increase in shear viscosity at low temperatures for the OPLS-AA force field using both methods and for the TraPPE-UA force field using the GK method. There is a small decrease in the values of TraPPE-UA using the MSD method.

**Fig. 4:**
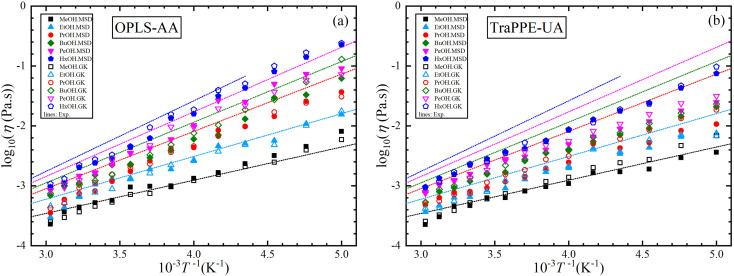
A comparison between OPLS-AA (a) and TraPPE-UA (b) force fields in predicted shear viscosity from MD simulations for pure 1-alkanol liquids by Green–Kubo (GK) and Einstein (MSD) methods in comparison to a correlation of linear form of the experimental data (lines).

For further comparison of different 1-alkanol liquids with each other, and to evaluate the validity of the Arrhenius relationship for the two force fields, Fig. 4 shows the relationship between the logarithm of the shear viscosity with the inverse of temperature. The two force fields are observed with the experimental data on the same sequence in increasing the shear viscosity with the increase in the number of methylene groups at all temperatures, but the OPLS-AA force field agrees in its values with the experimental values more. However, the mismatch does not have to be associated with the inadequacy of the amount of activation energy that is extracted from the slope of the straight line when using the Arrhenius relationship in its linear form.

**Fig. 5:**
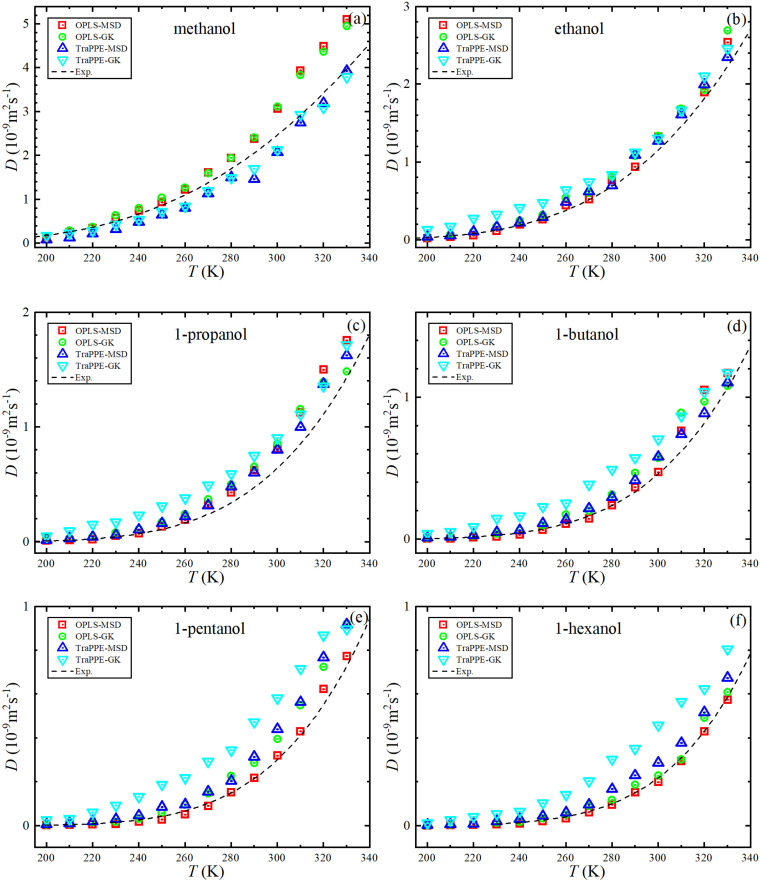
Temperature dependence of the predicted self-diffusion from MD simulations for pure 1-alkanol liquids (a–f) by Green–Kubo (GK) and Einstein (MSD) methods for OPLS-AA and TraPPE-UA force fields in comparison to above 260 K correlation of experimental data (dash lines).

Fig. 5 shows the dependence of the self-diffusion coefficient on temperature for the OPLS-AA and TraPPE-UA force fields using the Green–Kubo (GK) and Einstein (MSD) methods for 1-alkanol from methanol to 1-hexanol within a temperature range of 200–330 K compared to the experimental results. The two models using the two methods showed reliable results compared to the experiment and a correct prediction of the regression of self-diffusion values with decreasing temperature. TraPPE-UA showed better results for methanol using the two methods, while OPLS-AA showed an increase in values when temperatures increased. In ethanol, the results of the two models and using the two methods agreed with the experimental results to a considerable extent, except for an increase in TraPPE-UA using the Green–Kubo method at low temperatures (less than ∼260 K). For the other upper 1-alkanols, a concordance between the OPLS-AA and TraPPE-UA force fields when using the Green–Kubo and Einstein methods respectively, regardless of that, the OPLS-AA model showed the best results and a great agreement with the experimental results when using the Einstein method.

**Fig. 6:**
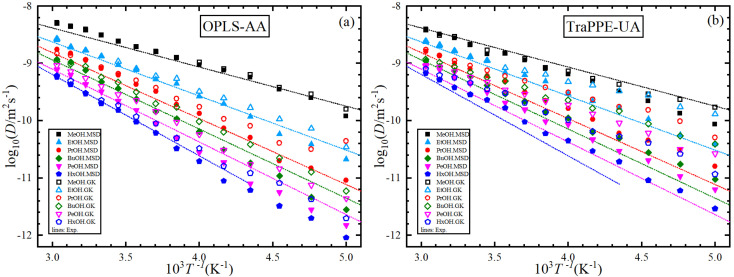
A comparison between OPLS-AA (a) and TraPPE-UA (b) force fields in predicted self-diffusion from MD simulations for pure 1-alkanol liquids by Green–Kubo (GK) and Einstein (MSD) methods in comparison to a correlation of linear form of the 265–330 K experimental data (solid lines).

We notice that the correlation curve fitting parameters in Fig. 5 and 6, listed in Table 2, pertain to temperatures above 260 K. These parameters show a better agreement with the original experimental data points compared to the correlation curve parameters for all temperatures (see ESI Fig. S2†). We also note that a comparison of MD self-diffusion results between this study and some results from other studies presented in the introduction is shown in Table S4 in the ESI.†

Fig. 6 shows the relationship between the logarithm of the self-diffusion with the inverse of temperature for all 1-alkanol liquids in our study. As for shear viscosity, the two force fields are observed with the experimental data on the same sequence at all temperatures but in decreasing the self-diffusion with the increase in the number of methylene groups, again, the OPLS-AA force field agrees in its values with the experimental values more, the activation energy of the self-diffusion was extracted from the slope of the straight line when using the linear form of the Arrhenius relationship. All The fitting parameters of the Arrhenius equation for both shear viscosity and self-diffusion and with both GK and MSD methods are reported in Table 3.

**Table 3:** Arrhenius equations fitting parameters of the MD data for the shear viscosity and self-diffusion of the OPLA-AA and TraPPE-UA force fields with both GK and MSD calculation methods

	*D*_0_ (10^−6^ m^2^ s^−1^)	*E*_*D*_ (kJ mol^−1^)	*η*_0_ (10^−6^ Pa s^−1^)	*E*_*η*_ (kJ mol^−1^)	*D*_0_ (10^−6^ m^2^ s^−1^)	*E*_*D*_ (kJ mol^−1^)	*η*_0_ (10^−6^ Pa s^−1^)	*E*_*η*_ (kJ mol^−1^)
	**OPLS-AA/MSD**				**TraPPE-UA/MSD**			
Methanol	1.34 ± 0.21	15.13 ± 0.33	2.30 ± 0.56	13.34 ± 0.51	1.26 ± 0.16	15.87 ± 0.27	5.93 ± 1.515	10.83 ± 0.54
Ethanol	4.27 ± 0.71	20.20 ± 0.35	1.79 ± 0.68	15.21 ± 0.79	1.43 ± 0.19	17.49 ± 0.27	3.58 ± 1.099	13.13 ± 0.64
1-Propanol	9.46 ± 1.98	23.33 ± 0.44	0.41 ± 0.12	19.33 ± 0.61	2.06 ± 0.27	19.63 ± 0.27	4.11 ± 1.087	13.29 ± 0.56
1-Butanol	20.86 ± 3.54	26.53 ± 0.36	0.88 ± 0.24	18.48 ± 0.58	1.78 ± 0.19	20.21 ± 0.23	4.09 ± 0.622	14.26 ± 0.32
1-Pentanol	17.50 ± 3.81	27.39 ± 0.46	0.33 ± 0.12	21.46 ± 0.78	2.10 ± 0.29	21.26 ± 0.29	5.30 ± 0.931	14.19 ± 0.37
1-Hexanol	14.05 ± 3.36	27.83 ± 0.50	0.17 ± 0.04	23.82 ± 0.54	2.87 ± 0.41	22.96 ± 0.30	1.65 ± 0.206	17.75 ± 0.26

	**OPLS-AA/GK**				**TraPPE-UA/GK**			
Methanol	0.87 ± 0.11	14.07 ± 0.27	2.52 ± 0.29	12.83 ± 0.24	0.45 ± 0.06	13.29 ± 0.29	2.30 ± 0.35	13.29 ± 0.32
Ethanol	1.53 ± 0.22	17.52 ± 0.30	2.13 ± 0.46	14.77 ± 0.45	0.17 ± 0.03	11.94 ± 0.34	3.70 ± 1.30	13.21 ± 0.73
1-Propanol	1.00 ± 0.44	17.79 ± 0.92	0.71 ± 0.19	18.21 ± 0.58	0.24 ± 0.04	13.78 ± 0.33	1.64 ± 0.27	15.73 ± 0.35
1-Butanol	4.54 ± 0.78	22.35 ± 0.36	0.25 ± 0.06	21.47 ± 0.52	0.23 ± 0.03	14.41 ± 0.28	2.96 ± 0.63	15.10 ± 0.45
1-Pentanol	4.42 ± 0.98	23.27 ± 0.46	0.67 ± 0.23	19.87 ± 0.72	0.29 ± 0.05	15.52 ± 0.33	2.63 ± 0.46	15.89 ± 0.37
1-Hexanol	3.66 ± 0.74	24.04 ± 0.43	0.19 ± 0.06	23.87 ± 0.66	0.45 ± 0.08	17.28 ± 0.40	1.24 ± 0.26	18.41 ± 0.45

The OPLS-AA force field shows better agreement with experimental self-diffusion values for higher 1-alkanols when using the MSD method because it employs a detailed atomic representation that captures molecular flexibility, hydrogen bonding, and intermolecular interactions more accurately. This force field is finely parameterized to reflect the unique properties of alcohols, allowing for precise modeling of their behavior. In contrast, the TraPPE-UA force field uses a simplified united atom approach, which, while generally effective, can overlook critical interactions and nuances, particularly for larger and more complex molecules. As a result, OPLS-AA can better handle the conformational dynamics and specific interactions of higher 1-alkanols, leading to more accurate predictions compared to TraPPE-UA.

**Fig. 7:**
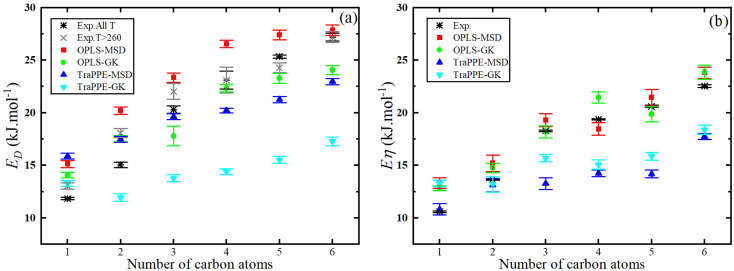
Number of 1-alkanol chain carbon atoms dependence of the predicted activation energy of the self-diffusion (a) and activation energy of the shear viscosity (b) from MD simulations by Green–Kubo (GK) and Einstein (MSD) methods for OPLS-AA and TraPPE-UA force fields in comparison to a correlation of experimental data listed in Table 2.

Fig. 7 shows that the activation energy of shear viscosity *E*_*η*_ and self-diffusion *E*_*D*_ increases with the number of carbon atoms in 1-alkanol liquids; specifically, for methanol *E*_*η*_ is ∼13% higher (OPLS-MSD), ∼7% higher (OPLS-GK), ∼7% lower (TraPPE-MSD), and ∼20% lower (TraPPE-GK) than experimental values, while for ethanol *E*_*η*_ is ∼10% higher (OPLS-MSD), ∼5% higher (OPLS-GK), ∼5% lower (TraPPE-MSD), and ∼16% lower (TraPPE-GK); for 1-propanol *E*_*η*_ is ∼10% higher (OPLS-MSD), 5∼% higher (OPLS-GK), ∼5% lower (TraPPE-MSD), and ∼14% lower (TraPPE-GK); and for higher 1-alkanols *E*_*η*_ is 8–10% higher (OPLS-MSD), 4–8% higher (OPLS-GK), 8–12% lower (TraPPE-MSD), and 16–20% lower (TraPPE-GK); for methanol *E*_*D*_ is ∼8% higher (OPLS-MSD), ∼8% lower (OPLS-GK), ∼15% lower (TraPPE-MSD), and ∼23% lower (TraPPE-GK) than experimental values, while for ethanol *E*_*D*_ is ∼13% higher (OPLS-MSD), ∼7% higher (OPLS-GK), ∼7% lower (TraPPE-MSD), and ∼20% lower (TraPPE-GK); for 1-propanol *E*_*D*_ is 11% higher (OPLS-MSD), ∼6% higher (OPLS-GK), ∼6% lower (TraPPE-MSD), and ∼17% lower (TraPPE-GK); and for higher 1-alkanols *E*_*D*_ is 9–12% higher (OPLS-MSD), 5–10% higher (OPLS-GK), 5–10% lower (TraPPE-MSD), and 15–20% lower (TraPPE-GK), indicating that OPLS-AA generally provides better agreement with experimental values for higher 1-alkanols, while TraPPE-UA performs better for methanol and ethanol, with both force fields showing consistent results between MSD and GK methods. Note that the united-atom force field will always underestimate the activation energies compared to the all-atom force field because the united atoms are smoother and exhibit less friction, which also explains why it overestimates the self-diffusion coefficient (Fig. 5) and underestimates shear viscosity (Fig. 3).

**Fig. 8:**
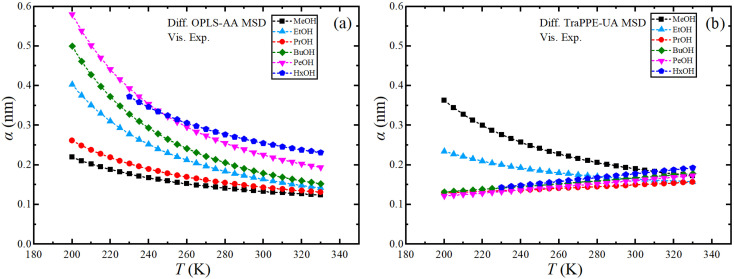
Temperature dependence of Stoke–Einstein effective hydrodynamic radius for pure 1-alkanol liquids by using MD-experimental data; MSD methods data of OPLS-AA (a) and TraPPE-UA (b) force fields were used for the self-diffusion and the experimental data for shear viscosity.

Finally, the effective hydrodynamic radius can be obtained using the data from MD simulations using the Stoke–Einstein relationship. Fig. S10 in ESI† shows the dependence of the effective hydrodynamic radius of the OPLS-AA and TraPPE-UA force fields on temperature, in which the average results from MD simulations using the two GK and MSD methods were used with the results of the self-diffusion of the two force fields by GK and MSD methods separately, fluctuation and a large difference between the methods and the force field appeared caused by the sensitivity of the calculation to the value of the effective hydrodynamic radius, so the effective hydrodynamic radius was determined using the experimental values of the shear viscosity and the self-diffusion coefficients determined from the MSDs calculated from the MD simulations as shown in Fig. 8. For more comparison, where we find the increment on the effective hydrodynamic radius at low temperatures.

## 5. Conclusion

The effect of the number of methylene groups of the 1-alkanol chain on the shear viscosity and self-diffusion of pure liquids was investigated by the MD simulations at temperatures range of 200–330 K, and by using the TraPPE-UA and the OPLS-AA force fields, and by the Green–Kubo and Einstein calculation methods. We compared the results of our simulations with the correlated experimental data. We found that the TraPPE-UA force field allows for the efficient study of large-size molecules, requiring much less computing time than the OPLS-AA force field due to its smaller number of atomic sites. At moderate temperatures (greater than 260 K), we observed that the results obtained from the TraPPE-UA are comparable to those obtained from the OPLS-AA force field. However, at lower temperatures (below 260 K) the TraPPE-UA force field was less useful for shear viscosity calculations, or when we used the GK method for the self-diffusion calculations. However, the TraPPE-UA force field performed well in predicting the properties of methanol and ethanol similarly to the OPLS-AA force field. Our analysis indicated that for higher 1-alkanols, the OPLS-AA force field showed better agreement with experimental self-diffusion values when using the MSD method. Additionally, the OPLS-AA force field demonstrated a better match with experimental shear viscosity values when using both the GK and MSD methods. TraPPE-UA and OPLS-AA force fields were both successful in predicting the trend of increased self-diffusion and decreased shear viscosity with the increment of temperature. Furthermore, both force fields correctly predicted that the increase in the 1-alkanol chain methylene groups number leads to an increase in viscosity values and a decrease in self-diffusion at all temperatures. The activation energy of self-diffusion and viscosity, as well as the effective hydrodynamic radius, were calculated, and both force fields success in predicting the increase in the activation energy of self-diffusion and viscosity as the number of 1-alkanol methylene groups increased. However, for methanol, the TraPPE-UA force field demonstrated an advantage in calculating the activation energy for shear viscosity using the MSD method and for self-diffusion using the GK method, and for other 1-alkanols, the OPLS-AA force field performed better in calculating the activation energies of self-diffusion and shear viscosity using both the MSD and GK methods. Calculating the effective hydrodynamic radius required the values of the self-diffusion and the shear viscosity coefficients at a certain temperature, the results of average shear viscosity were taken from the MSD and GK methods and plotted with the diffusivity results, The GK method for self-diffusion resulted in more stable values of the effective hydrodynamic radius than the MSD method for both force fields, as the MSD method showed that the effective hydrodynamic radius values increased at low temperatures with relative stability at temperatures greater than about 270 K. It was also observed when the experimental shear viscosity results were combined with the MSD results of self-diffusion from the simulation it will give the best-correlated results. Finally, the Stoke–Einstein relation was found to be valid for pure 1-alkanol liquids, but with a temperature-dependent effective hydrodynamic radius, particularly at low temperatures.

## Data availability

All data are available upon request.

## Conflicts of interest

There are no conflicts to declare.

## Supplementary Material

RA-014-D4RA03494E-s001
