# Supplementary material for: Self-diffusion and shear viscosity of pure 1-alkanol unary system: molecular dynamics simulation and review of experimental data
Source: RSC Adv. 2024 Jul 22;14(32):22947–61. doi: 10.1039/d4ra03494e (PMC11261341; doi:10.1039/d4ra03494e)

Electronic Supplementary Material (ESI) for RSC Advances.
This journal is © The Royal Society of Chemistry 2024

Supporting Information

Self-diffusion and shear viscosity of pure 1-alkanol unary system: molecular dynamics simulation and review of experimental data

Adnan Jaradat[1], Rakan Al-Salman[1] and Abdalla Obeidat*[1]

[1]Department of Physics, Jordan University of Science and Technology, Irbid, Jordan

*Corresponding author: aobeidat@just.edu.jo

Comparison between Linear and Non-Linear Models in Extracting Activation Energy

To extract activation energy from experimental data, both linear models (Arrhenius equation) and non-linear models (such as the Vogel-Fulcher-Tammann (VFT) equation) can be used. The choice of the appropriate model depends on the nature of the system and the behavior of the data.

The Arrhenius equation is simple, widely used, and straightforward to apply and interpret, This model has several advantages. Firstly, it is easy to apply and interpret. Secondly, when plotting $\log_{10}(D,\eta)$ versus $1/T$, it produces a straight line, making it easy to extract the activation energy from the slope. Thirdly, it is well-documented and understood, facilitating comparisons with existing literature. However, the Arrhenius equation has some disadvantages. It assumes a single activation energy over the entire temperature range, which may not be valid for all systems. Additionally, it may not accurately describe systems with complex temperature dependence.

On the other hand, the Vogel-Fulcher-Tammann (VFT) equation is an example of a non-linear model. The VFT equation is better suited for systems where diffusion or viscosity changes significantly with temperature, such as glass-forming liquids. It can capture more complex behaviors, providing a more accurate representation of the data. However, it is more complex to apply and interpret. The relationship is non-linear, making it harder to extract and interpret parameters. Additionally, it is less common, so there may be fewer studies for comparison.

When choosing the appropriate model, several practical considerations should be considered. If the data plot as a straight line on an Arrhenius plot $\log10(D,\eta)$ versus $1/T$, the linear model is likely sufficient and appropriate. If there are significant deviations from linearity, a non-linear model might be necessary. For narrow temperature ranges, the Arrhenius equation often works well. For broader ranges or systems approaching a glass transition or other critical points, non-linear models might be more accurate. For simple systems with well-defined activation energies, linear models are appropriate. For systems with complex interactions or multiple processes contributing to diffusion, non-linear models provide a better fit.

For our study on the self-diffusion and shear viscosity of 1-alkanols from methanol to 1-hexanol, starting with the Arrhenius equation is reasonable. The fits shown in all the plots demonstrate the linear trend within the temperature range used in this study for both experimental and simulation results (see Figure S1 for example). Any deviations from linearity are very small. Additionally, the additional parameters in non-linear equations significantly affect the extraction of activation energies

from the fitting process, especially given the use of multiple sources with noticeable differences in values. This makes non-linear fitting impractical for our study. Moreover, many studies have successfully used linear fitting for primary alcohol systems with simple structures, demonstrating the efficiency of linear fitting in extracting activation energies for self-diffusion and viscosity

In conclusion, while both linear and non-linear models have their merits, the linear model provides a practical and effective approach for our specific study on 1-alkanols, ensuring simplicity and accuracy in determining activation energy.

Figure S1: The dependence of each experimental reference self-diffusion on the temperature for methanol (MeOH), ethanol (EtOH), 1-propanol (PrOH), 1-butanol (BuOH), 1-pentanol (PeOH), and 1-hexanol (HxOH). Solid lines are the linear fitting using the Arrhenius equation.

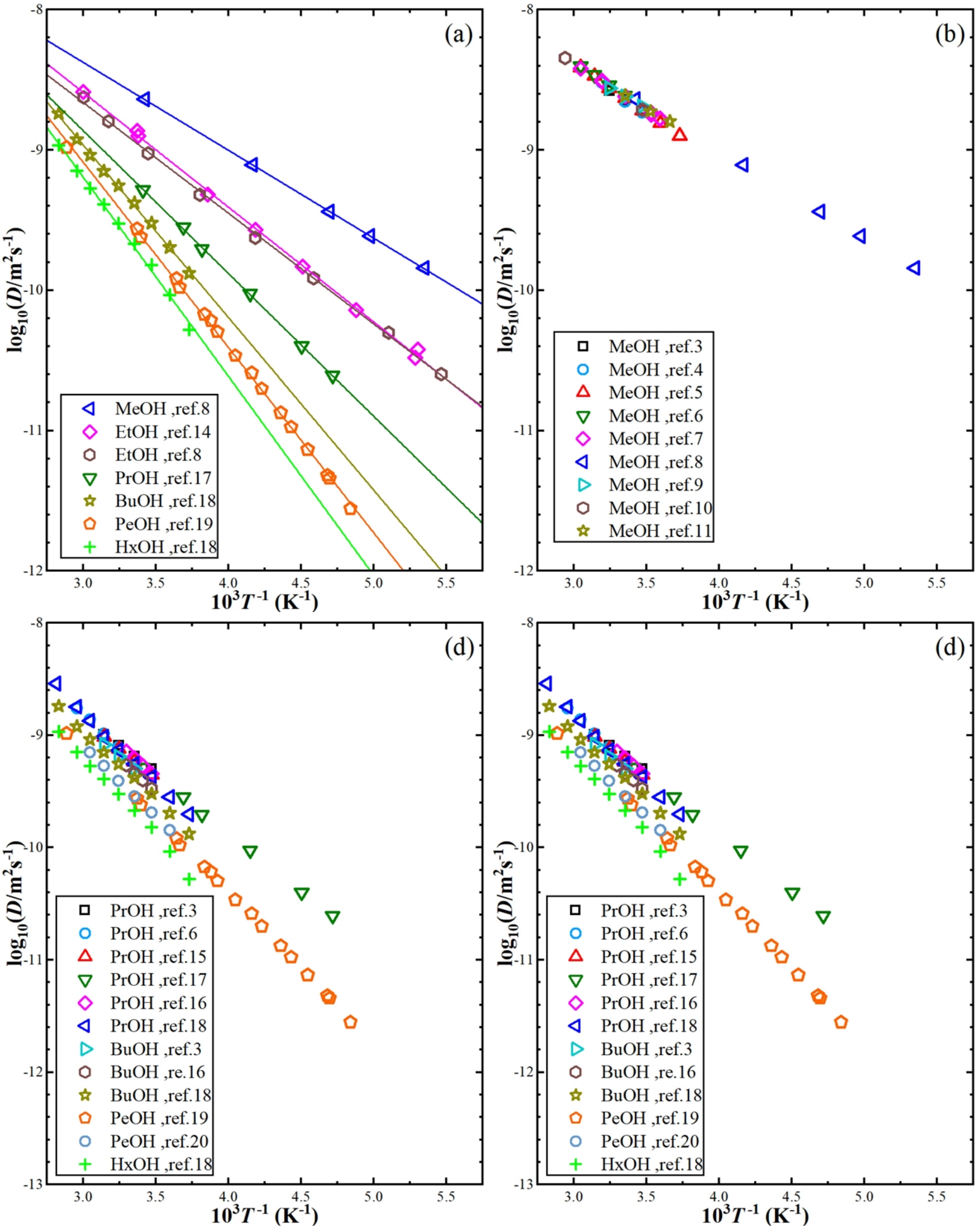

Figure S2: The dependence of the experimental self-diffusion (Left) and shear viscosity (Right) on the temperature. methanol (MeOH), ethanol (EtOH), 1-propanol (PrOH), 1-butanol (BuOH), 1-pentanol (PeOH), and 1-hexanol (HxOH). Solid curves represent the exponential fitting using all points, and dash line for exponential fitting using points within the temperature range of (265-330K) by using the Arrhenius equation.

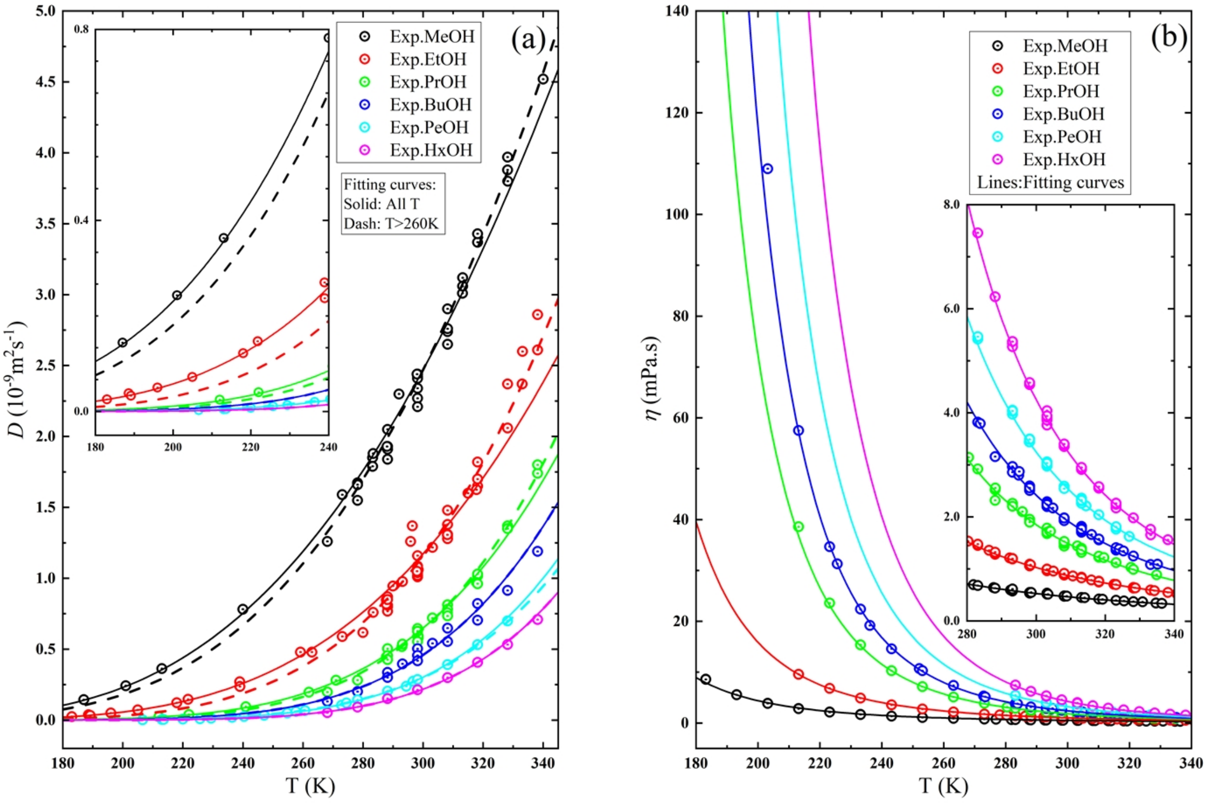

Figure S3: The relationship between shear viscosity and temperature divided by self-diffusion for each 1-alkanol system.

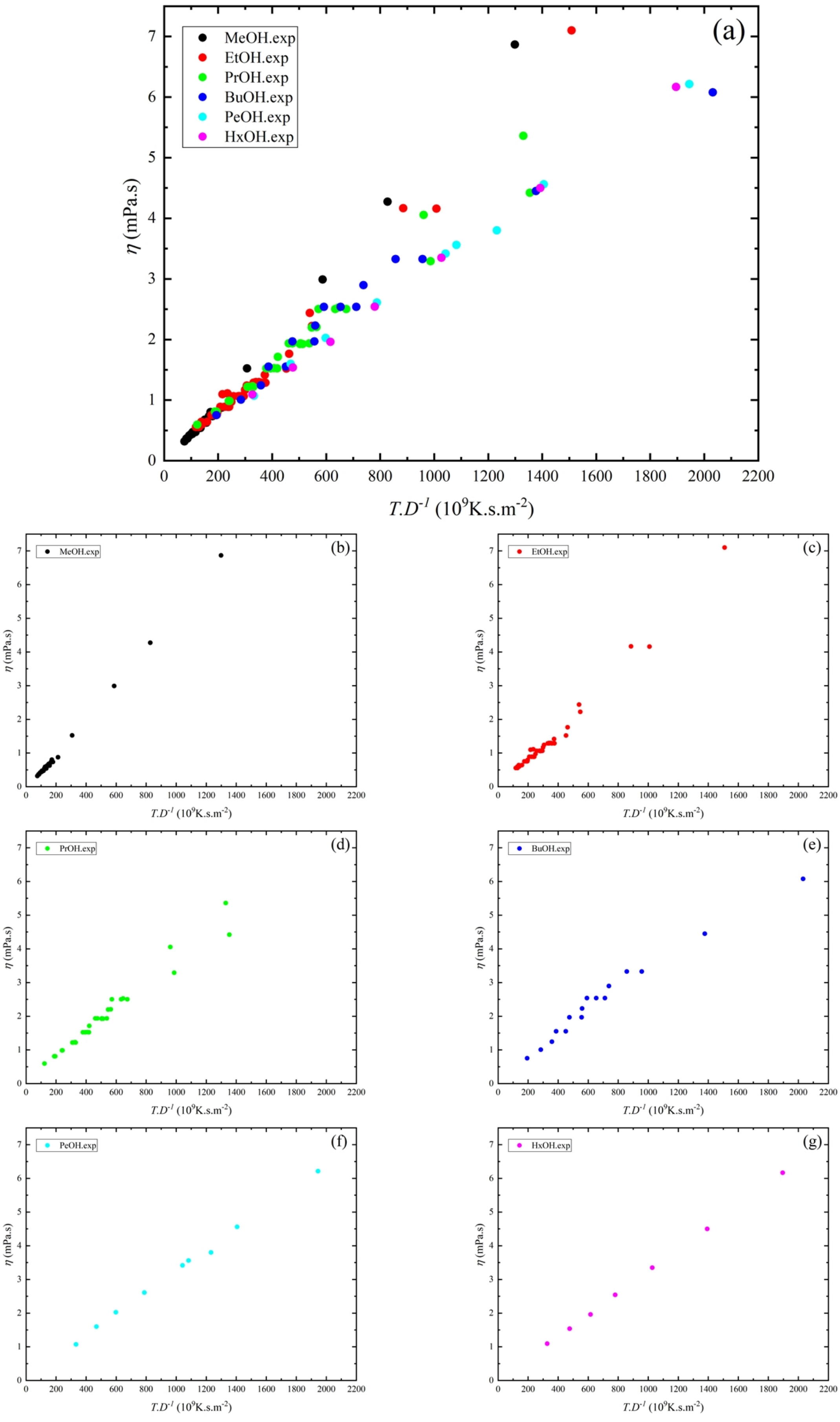

Figure S4: OPLS-AA and TraPPE-UA force fields Single molecule snapshots for 1-alkanol from methanol to 1-hexanol.

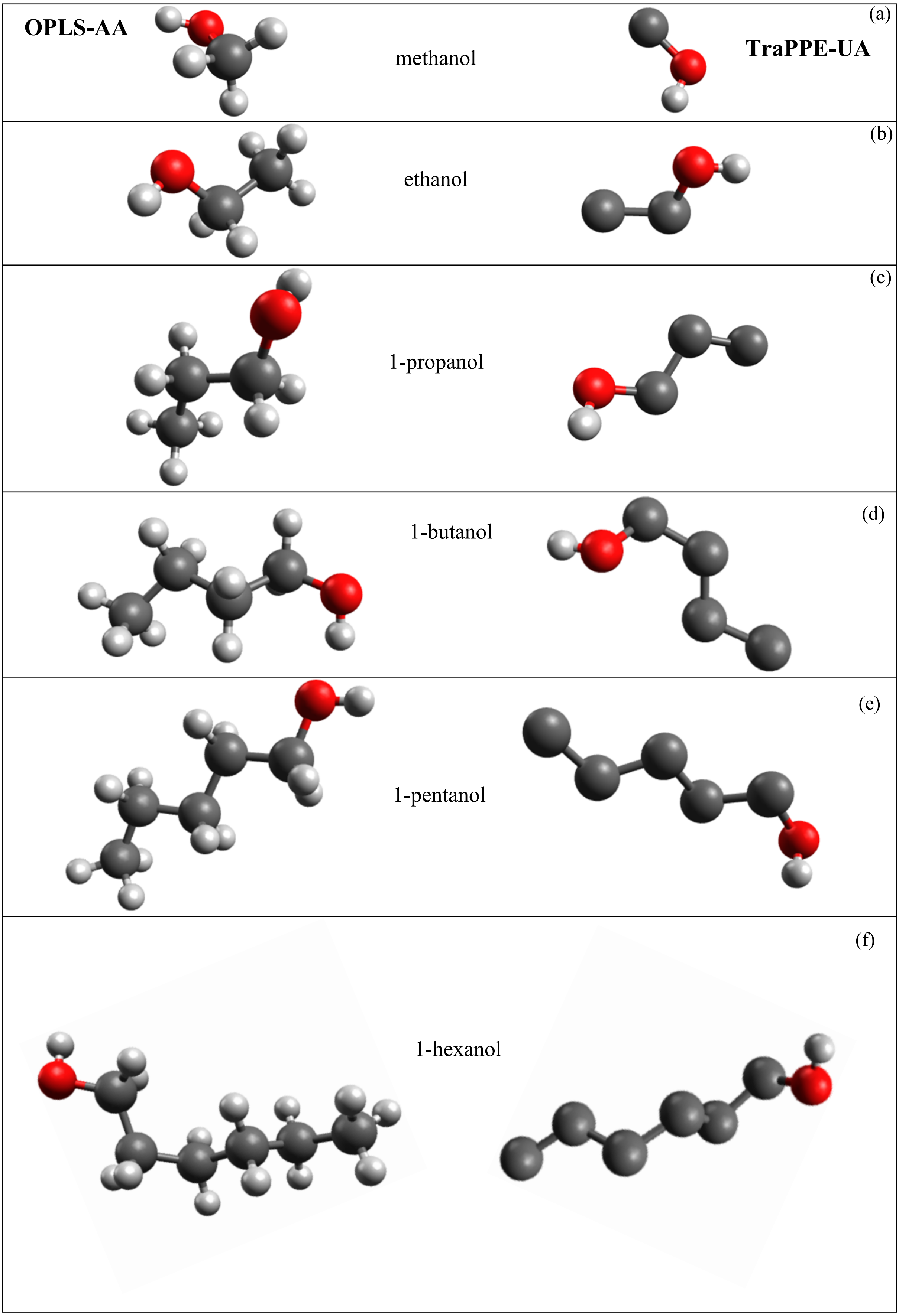

Table S1: Lennard-Jones parameters and the geometries of the TraPPE-UA and OPLS-AA force field. The harmonic and torsional potentials are used to control bond angle bending and to control the dihedral rotations around bonds, and the bonded potential can be written as:

$$U_b = \sum_{angles} \frac{k_\theta}{2}(\theta - \theta_{eq})^2 + \sum_{dihedrals} \sum_{i=1}^{3} T_i[1 - (-1)^i \cos(i\varphi)]$$

where k_θ is the spring constant, θ_{eq} is the equilibrium angle, φ is s the angle between the planes of the angles, and F_i are the Fourier coefficients.

TraPPE-UA non-bonded parameters and geometries

Atom (pseudo)	Type	σ (nm)	ε (kJ/mol)	q (e)
CH_3	$[CH_3]$-O-H	0.375	0.814815094	0.265
CH_3	$[CH_3]$-CHx	0.375	0.814815094	0
CH_2	CHx-$[CH_2]$-O-H	0.395	0.382464228	0.265
O	CHx-[O]-H	0.302	0.773242895	-0.7
H	O-[H]	0	0	0.435

Bond type	Bond-length (nm)	Angle type	θ (degrees)
CHx-CHy	0.154	CHx-(CH_2)-CHy	114
CHx-OH	0.143	CHx-(CHy)-OH	109.47
O-H	0.0945	CHx-(O)-H	108.5

**Bend type**	**k_θ(kJ.mol^{-1})**	**Torsion type**	**T_1(kJ.mol^{-1})**	**T_2(kJ.mol^{-1})**	**T_3(kJ.mol^{-1})**
CHx-(CH_2)-CHy	519.6539	CHx-(CH_2) -(CH_2)-CHy	1.5486	-0.2974	3.4516
CHx-(CH_2)-OH	419.0489	CHx-(CH_2) -(CH_2)-O	0.7704	-0.2327	3.3583
CHx-(O)-H	460.6212	CHx-(CH_2)-(O)-H	0.9152	-0.1272	0.8197

OPLS-AA non-bonded parameters and geometries

Atom (pseudo)	Type	σ (nm)	ε (kJ/mol)	q (e)
H	$C(H)_3$-CH_2-R	0.25	0.12552	0.06
H	CHx-$C(H)_2$-CHy	0.25	0.12552	0.06
C	$(C)H_3$-C	0.35	0.276144	-0.18
C	CHx-$(C)H_2$-Chy	0.35	0.276144	-0.12
C	(C)Hx-OH	0.35	0.276144	0.145
O	C-(O)-H	0.312	0.71128	-0.683
H	O-(H)	0	0	0.418
H	$C(H)_3$-OH	0.25	0.12552	0.04

Bond type	Bond-length (nm)	Angle type	θ (degrees)
H-O	0.0945	C-C-H	110.7
C-C	0.1529	H-C-O	109.5
C-O	0.141	C-O-H	108.5
C-H	0.109	C-C-C	112.7
		H-C-H	107.8
		C-C-O	109.5

**Bend type**	**k_θ(kJ.mol^{-1})**	**Torsion type**	**T_1(kJ.mol^{-1})**	**T_2(kJ.mol^{-1})**	**T_3(kJ.mol^{-1})**
C-C-C	488.273	C-C-C-O	3.5794	-1.046	1.386996
C-C-O	418.4	C-C-O-HO	-0.74475	-0.36401	1.029264
C-O-Ho	460.24	H-C-O-HO	0	0	0.9414
H-C- H	276.144	H-C-C-O	0	0	0.97905
H-C- O	292.88	H-C-C-H	0	0	0.6276
		C-C-C-H	0	0	0.6276

Figure S5: initial model diagram for molecular dynamics simulation.

System Setup

- Software: GROMACS 2018.8
- Molecules: 1000 for self-diffusion, 500 for shear viscosity
- Box Sizes: 4.1 nm^3 to 5.9 nm^3
- Periodic Boundary Conditions

Energy Minimization

- Algorithm: Steepest Descent
- Maximum Steps: 200,000
- Energy Step: 2×10^{-5} kJ/mol

Equilibration

- Step 1: NPT Equilibration
  - Algorithm: Parrinello-Rahman
  - Duration: 2 ns
  - Reference Pressure: 1 bar
- Step 2: NVT Equilibration
  - Algorithm: Nosé-Hoover
  - Duration: 2 ns
  - Conditions: Constant Temperature, Fixed Size
- Time Step: 2 fs
- Boundary Conditions: Periodic
- Integrator: Leap-Frog
- Bond Correction: LINCS Algorithm
- Cut-off Radii: 1.3 nm (OPLS-AA), 1.4 nm (TraPPE-UA)

Data Collection and Analysis

- Self-Diffusion Coefficient
- Methods: Green-Kubo, Einstein
- Data Collection :
- Green-Kubo: 100 ps, frames every step
- Einstein: 6 ns, frames every 100 steps
- Repetitions :
- Green-Kubo: 8 times
- Einstein: 16 times
- Shear Viscosity :
- Methods: Green-Kubo, Einstein
- Repetitions: 16 times
- Data: Pressure Tensors
- Analysis: Time average, standard deviation

Table S2: The box sizes and bulk densities for the molecular systems used in this work. These include 1000 molecules used for self-diffusion MD simulations and 500 molecules used for shear viscosity MD simulations. All 1-alkanols used in these simulations were governed by the TraPPE-UA and OPLS-AA force fields.

		**Self-diffusion (1000 molecules)**				**Shear viscosity (500 molecules)**				
		OPLS-AA		TraPPE-UA		OPLS-AA		TraPPE-UA		Exp.
	T (K)	d_x (nm)	ρ (kg/m^3)	d_x (nm)	ρ (kg/m^3)	d_x (nm)	ρ (kg/m^3)	d_x (nm)	ρ (kg/m^3)	ρ (kg/m^3)
**methanol**	200	3.912±0.007	888.8±2.5	3.926±0.008	878.9±2.5	3.113±0.009	881.8±7.5	3.129±0.009	868.5±7.6	880.18±0.31
	210	3.943±0.008	867.6±2.5	3.933±0.008	874.7±2.8	3.129±0.008	868.4±6.6	3.139±0.009	860.4±7.3	870.28±0.29
	220	3.946±0.010	866.2±3.2	3.941±0.008	869.1±2.6	3.145±0.009	855.2±7.1	3.148±0.011	852.5±9.2	860.51±0.26
	230	3.984±0.009	841.1±2.9	3.975±0.007	846.9±2.2	3.154±0.008	847.9±6.2	3.158±0.010	844.5±8.0	850.87±0.23
	240	3.996±0.008	833.7±2.6	3.974±0.010	847.5±3.1	3.164±0.008	839.9±6.6	3.168±0.010	836.7±8.3	841.33±0.19
	250	4.008±0.010	826.6±3.1	3.997±0.012	833.3±3.8	3.188±0.009	821.0±7.1	3.180±0.012	827.5±9.2	831.86±0.15
	260	4.021±0.010	818.3±2.9	3.992±0.011	836.6±3.5	3.212±0.010	802.8±7.8	3.191±0.014	818.6±11.2	822.44±0.12
	270	4.040±0.012	806.7±3.7	4.024±0.011	816.3±3.4	3.196±0.011	814.9±8.5	3.200±0.010	811.5±7.6	813.05±0.09
	280	4.049±0.010	801.2±3.0	4.049±0.016	801.3±4.6	3.235±0.012	785.8±8.7	3.230±0.013	789.1±10.0	803.66±0.07
	290	4.067±0.012	790.9±3.5	4.036±0.013	809.4±3.8	3.250±0.012	774.9±9.0	3.221±0.015	796.3±11.5	794.26±0.06
	300	4.109±0.012	767.2±3.3	4.062±0.014	793.7±4.0	3.235±0.014	785.8±10.1	3.233±0.014	787.2±10.3	784.81±0.07
	310	4.141±0.014	749.3±3.9	4.118±0.016	762.1±4.5	3.266±0.013	763.6±9.4	3.247±0.012	777.2±8.9	775.30±0.10
	320	4.153±0.015	743.0±4.1	4.101±0.013	771.6±3.7	3.296±0.013	742.9±8.8	3.278±0.014	755.3±9.7	765.70±0.15
	330	4.152±0.018	743.3±4.9	4.125±0.014	757.9±3.9	3.310±0.017	733.5±11.7	3.296±0.018	743.3±12.2	755.99±0.23
**ethanol**	200	4.402±0.007	896.8±2.1	4.443±0.007	872.4±2.1	3.489±0.007	900.6±5.8	3.533±0.009	867.5±6.3	870.01±0.16
	210	4.420±0.008	886.2±2.4	4.446±0.009	870.5±2.5	3.518±0.008	878.5±5.9	3.537±0.008	864.6±5.9	860.83±0.14
	220	4.423±0.008	883.8±2.3	4.479±0.009	851.1±2.5	3.546±0.008	857.9±5.9	3.541±0.010	861.7±7.4	851.87±0.12
	230	4.454±0.007	865.9±2.1	4.495±0.008	842.5±2.3	3.544±0.010	859.3±7.0	3.560±0.009	847.5±6.6	843.10±0.11
	240	4.471±0.008	855.9±2.3	4.513±0.010	832.5±2.7	3.543±0.010	860.1±7.4	3.580±0.011	833.7±7.5	834.47±0.10
	250	4.482±0.009	849.5±2.5	4.515±0.008	831.4±2.3	3.560±0.011	847.8±8.0	3.588±0.011	828.0±8.0	825.95±0.09
	260	4.528±0.008	823.9±2.2	4.548±0.009	813.2±2.6	3.576±0.010	836.5±7.0	3.596±0.010	822.4±7.0	817.50±0.08
	270	4.525±0.012	825.9±3.3	4.555±0.011	809.4±2.8	3.603±0.012	817.8±8.1	3.628±0.013	801.1±8.3	809.07±0.08
	280	4.550±0.010	812.0±2.7	4.547±0.012	813.5±3.2	3.622±0.014	805.0±9.2	3.618±0.013	807.9±8.7	800.63±0.08
	290	4.569±0.012	801.9±3.2	4.597±0.010	787.5±2.5	3.631±0.011	799.0±7.1	3.648±0.011	787.6±7.2	792.14±0.09
	300	4.593±0.012	789.7±3.1	4.601±0.012	785.6±3.0	3.631±0.014	799.0±9.1	3.671±0.014	773.2±9.0	783.55±0.10
	310	4.617±0.014	777.3±3.6	4.635±0.013	768.3±3.3	3.661±0.015	779.5±9.9	3.668±0.013	775.2±8.5	774.84±0.11
	320	4.620±0.013	775.7±3.4	4.665±0.015	753.3±3.6	3.684±0.015	765.0±9.5	3.722±0.014	741.7±8.4	765.95±0.13
	330	4.663±0.017	754.6±4.1	4.656±0.014	757.8±3.4	3.716±0.016	745.4±9.5	3.713±0.016	747.2±9.5	756.85±0.15
**1-propanol**	200	4.880±0.010	858.5±2.6	4.838±0.006	881.4±1.6	3.828±0.010	889.5±6.7	3.860±0.006	867.7±4.2	878.81±0.22
	210	4.830±0.006	885.6±1.7	4.864±0.007	867.3±1.9	3.844±0.008	878.4±5.5	3.870±0.008	860.6±5.7	870.39±0.21
	220	4.830±0.007	885.8±2.0	4.889±0.008	853.8±2.1	3.860±0.008	867.6±5.7	3.881±0.009	853.6±6.0	862.07±0.21
	230	4.871±0.007	863.6±1.9	4.888±0.009	854.7±2.3	3.869±0.008	861.5±5.4	3.891±0.008	846.8±5.4	853.85±0.20
	240	4.867±0.008	865.4±2.2	4.906±0.010	845.3±2.6	3.878±0.007	855.5±5.0	3.902±0.011	840.1±7.0	845.73±0.19
	250	4.893±0.008	852.0±2.0	4.931±0.008	832.4±2.1	3.891±0.012	847.0±7.9	3.909±0.009	835.5±6.0	837.70±0.18
	260	4.907±0.012	844.5±3.1	4.942±0.011	826.7±2.7	3.904±0.010	838.6±6.5	3.916±0.010	831.0±6.5	829.74±0.18
	270	4.938±0.010	828.6±2.5	4.957±0.011	819.1±2.8	3.926±0.011	824.5±7.3	3.944±0.011	813.4±7.1	821.84±0.17
	280	4.953±0.014	821.5±3.6	4.986±0.013	805.2±3.2	3.964±0.014	801.0±8.7	3.965±0.010	800.6±6.2	813.96±0.16
	290	4.976±0.011	809.8±2.8	4.995±0.013	800.9±3.1	3.963±0.016	801.7±9.5	3.966±0.012	800.1±7.3	806.08±0.15
	300	4.993±0.015	801.9±3.6	5.008±0.013	794.5±3.1	3.983±0.016	789.6±9.6	3.985±0.012	788.7±7.2	798.16±0.15
	310	5.024±0.016	786.8±3.8	5.020±0.012	789.0±2.9	4.019±0.017	768.6±10.0	3.982±0.013	790.4±7.7	790.15±0.14
	320	5.031±0.018	783.5±4.3	5.052±0.012	773.8±2.9	4.038±0.023	757.8±13.2	3.998±0.014	780.9±8.2	782.00±0.13
	330	5.023±0.022	787.2±5.2	5.063±0.014	768.7±3.2	4.102±0.027	722.9±14.3	4.019±0.014	768.7±7.8	773.66±0.13
**1-butanol**	200	5.172±0.009	889.4±2.4	5.193±0.005	879.1±1.3	4.105±0.007	889.7±4.8	4.125±0.008	877.0±5.0	881.32±0.42
	210	5.174±0.006	888.7±1.7	5.208±0.006	871.4±1.5	4.129±0.009	874.2±6.1	4.136±0.007	869.8±4.7	873.06±0.37
	220	5.200±0.008	875.3±2.0	5.216±0.008	867.3±1.9	4.153±0.008	859.2±4.8	4.147±0.008	862.6±5.3	865.03±0.32
	230	5.214±0.009	868.3±2.3	5.247±0.007	852.1±1.7	4.164±0.009	852.4±5.4	4.158±0.011	856.0±6.8	857.19±0.29
	240	5.229±0.007	861.1±1.9	5.244±0.008	853.7±2.0	4.175±0.009	845.7±5.4	4.169±0.009	849.4±5.5	849.51±0.25
	250	5.250±0.010	850.4±2.5	5.272±0.009	840.1±2.0	4.195±0.011	833.6±6.5	4.176±0.009	845.0±5.2	841.93±0.23
	260	5.271±0.011	840.5±2.6	5.282±0.010	835.3±2.4	4.215±0.015	821.8±8.7	4.183±0.009	840.5±5.4	834.43±0.21
	270	5.283±0.014	834.6±3.3	5.314±0.011	820.3±2.7	4.234±0.012	810.8±7.2	4.218±0.011	820.0±6.3	826.96±0.19
	280	5.302±0.013	825.7±3.2	5.324±0.011	815.5±2.5	4.237±0.013	809.1±7.5	4.219±0.013	819.7±7.9	819.49±0.17
	290	5.339±0.017	808.8±3.8	5.348±0.010	804.8±2.3	4.268±0.016	791.6±8.9	4.231±0.014	812.3±8.2	811.98±0.17
	300	5.342±0.016	807.2±3.6	5.347±0.012	805.3±2.8	4.284±0.017	782.7±9.5	4.267±0.013	792.3±7.4	804.39±0.17
	310	5.392±0.019	785.0±4.2	5.377±0.009	791.8±2.0	4.372±0.017	736.4±8.5	4.263±0.011	794.2±6.3	796.68±0.17
	320	5.384±0.025	788.6±5.6	5.372±0.011	794.1±2.5	4.318±0.017	764.4±9.1	4.288±0.013	780.6±7.3	788.82±0.18
	330	5.379±0.028	790.6±6.3	5.367±0.011	796.1±2.5	4.361±0.022	742.0±11.6	4.303±0.016	772.5±8.9	780.76±0.20
**1-pentanol**	200	5.492±0.008	883.8±1.9	5.503±0.007	878.6±1.8	4.364±0.008	880.6±5.0	4.361±0.007	882.4±4.5	-
	210	5.486±0.009	886.7±2.3	5.512±0.007	874.0±1.6	4.371±0.006	876.4±3.7	4.374±0.009	874.9±5.5	875.72±0.34
	220	5.515±0.008	872.8±2.0	5.539±0.006	861.4±1.4	4.378±0.010	872.2±6.3	4.386±0.007	867.5±4.1	868.27±0.34
	230	5.514±0.010	873.0±2.4	5.541±0.008	860.3±2.0	4.398±0.010	860.4±6.0	4.403±0.008	857.2±4.8	860.92±0.33
	240	5.528±0.011	866.7±2.5	5.564±0.008	849.8±1.8	4.418±0.012	848.7±6.7	4.421±0.009	847.0±5.4	853.64±0.33
	250	5.552±0.012	855.1±2.9	5.584±0.008	840.6±1.9	4.428±0.008	843.0±4.9	4.436±0.009	838.5±5.2	846.40±0.31
	260	5.574±0.012	845.4±2.7	5.583±0.010	841.2±2.2	4.438±0.015	837.3±8.4	4.451±0.009	830.0±5.1	839.17±0.30
	270	5.592±0.013	836.9±2.9	5.608±0.009	829.7±2.0	4.464±0.012	822.8±6.9	4.457±0.010	826.8±5.4	831.94±0.28
	280	5.622±0.012	823.6±2.7	5.611±0.009	828.5±1.9	4.491±0.012	808.0±6.7	4.464±0.011	822.5±6.0	824.68±0.26
	290	5.633±0.016	819.1±3.5	5.643±0.008	814.6±1.8	4.495±0.011	805.8±6.1	4.469±0.011	820.2±6.3	817.35±0.23
	300	5.653±0.022	810.1±4.8	5.653±0.009	810.5±1.9	4.529±0.018	787.8±9.4	4.491±0.011	807.9±6.1	809.93±0.21
	310	5.679±0.021	799.3±4.4	5.676±0.012	800.7±2.5	4.544±0.014	780.1±7.4	4.492±0.013	807.6±7.2	802.41±0.20
	320	5.701±0.029	789.9±6.0	5.695±0.011	792.4±2.2	4.583±0.019	760.3±9.4	4.502±0.012	801.9±6.5	794.74±0.19
	330	5.718±0.018	782.9±3.6	5.672±0.012	802.2±2.5	4.620±0.021	742.2±10.1	4.531±0.012	786.8±6.4	786.91±0.18
**1-hexanol**	200	5.761±0.009	887.3±2.1	5.753±0.006	891.2±1.4	4.576±0.007	885.2±4.3	4.573±0.007	887.2±4.1	-
	210	5.766±0.007	884.8±1.7	5.759±0.007	888.1±1.5	4.586±0.008	879.4±4.8	4.582±0.007	881.5±4.1	-
	220	5.764±0.008	886.0±1.8	5.786±0.007	875.7±1.6	4.596±0.009	873.7±5.4	4.592±0.008	875.8±4.8	-
	230	5.790±0.009	874.0±2.0	5.812±0.008	864.3±1.8	4.603±0.010	869.7±5.6	4.613±0.007	864.2±4.0	861.78±0.51
	240	5.802±0.009	868.4±2.0	5.828±0.009	857.0±1.9	4.610±0.011	865.8±6.2	4.633±0.010	852.8±5.8	855.25±0.37
	250	5.831±0.010	855.9±2.2	5.835±0.008	854.1±1.8	4.639±0.009	849.6±5.0	4.638±0.009	850.2±5.0	848.66±0.25
	260	5.873±0.012	837.3±2.5	5.834±0.010	854.4±2.3	4.669±0.012	833.4±6.5	4.643±0.008	847.6±4.6	841.98±0.16
	270	5.895±0.012	828.0±2.6	5.848±0.010	848.3±2.3	4.688±0.014	823.3±7.4	4.652±0.011	842.8±5.8	835.20±0.14
	280	5.888±0.013	830.9±2.9	5.883±0.011	833.3±2.3	4.698±0.013	818.0±6.7	4.685±0.011	825.1±5.7	828.32±0.13
	290	5.930±0.015	813.3±3.0	5.909±0.010	822.0±2.2	4.728±0.016	802.6±8.2	4.695±0.010	819.5±5.5	821.33±0.10
	300	5.986±0.018	791.0±3.5	5.899±0.011	826.4±2.4	4.747±0.016	793.0±8.1	4.696±0.011	818.9±5.6	814.21±0.10
	310	5.997±0.019	786.7±3.7	5.960±0.011	801.3±2.2	4.761±0.019	786.0±9.5	4.711±0.011	811.3±5.7	806.96±0.10

320	6.022±0.024	776.8±4.7	5.940±0.011	809.4±2.3	4.808±0.015	763.2±7.0	4.728±0.014	802.7±7.2	799.56±0.12
330	6.075±0.023	756.5±4.2	5.960±0.012	801.5±2.5	4.818±0.022	758.4±10.6	4.738±0.012	797.6±6.2	792.00±0.12

Figure S6: Examples of MSD and VACF profiles for 1-alkanols at 200 K and 330 K.

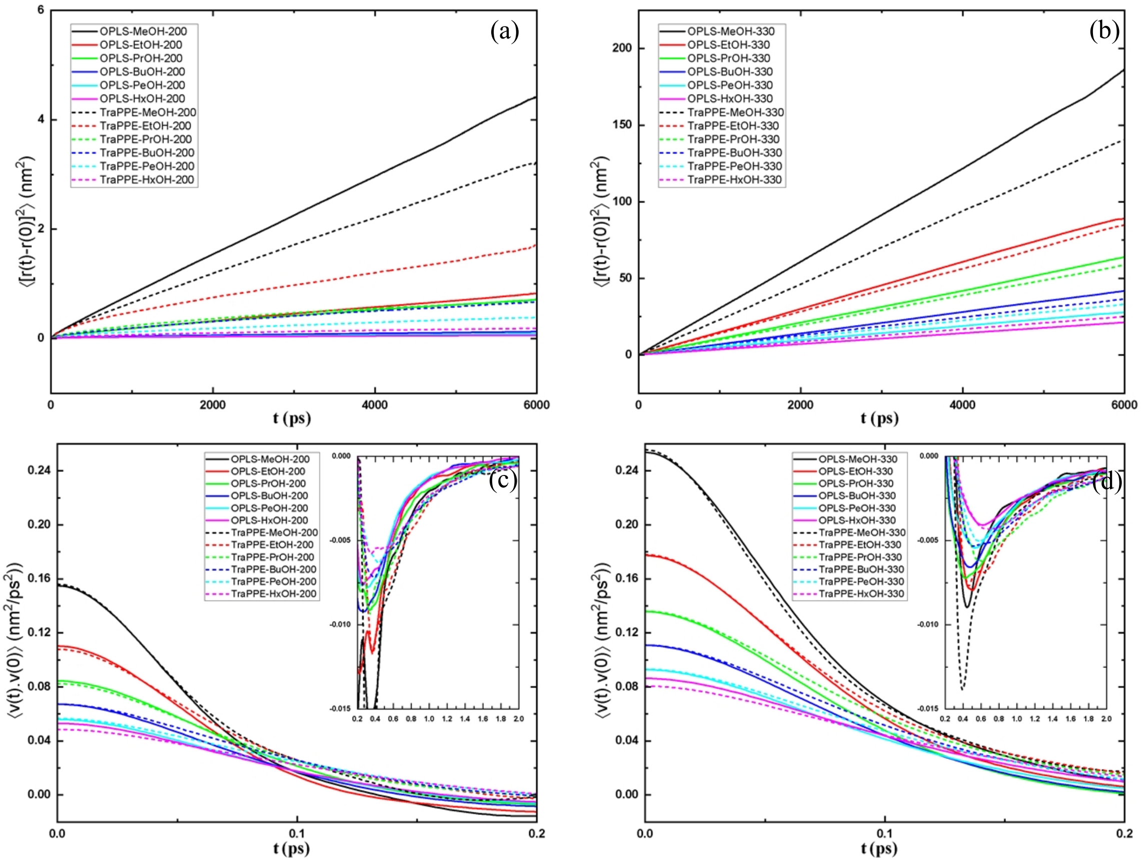

Figure S7: Trials of the self-diffusion by the GK method.

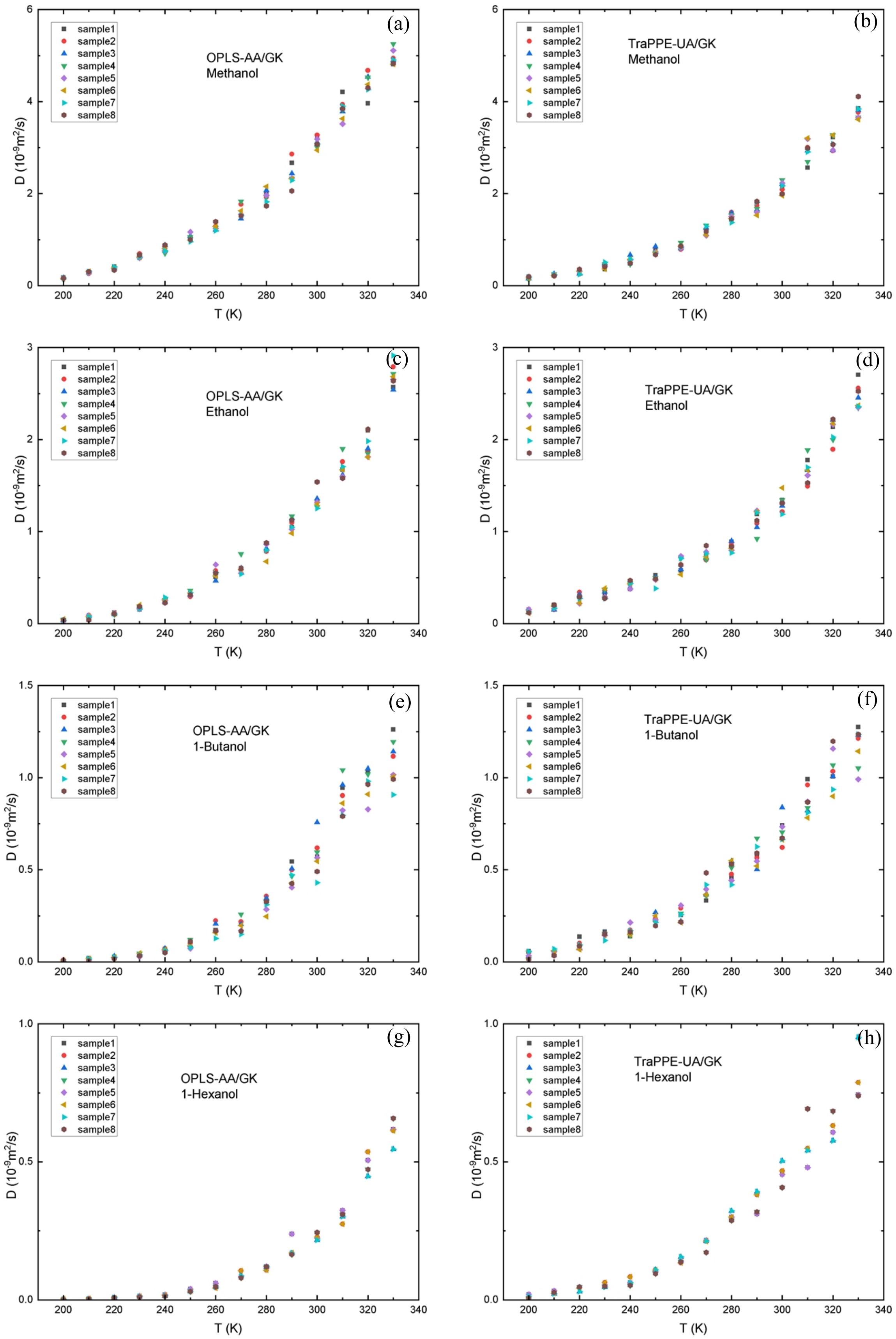

Figure S8: The average time dependence integral values of shear viscosity for methanol, 1-propanol, and 1-pentanol at 200, 270, and 330 K by GK and MSD methods and for both TraPPE-UA and OPLS-AA force fields.

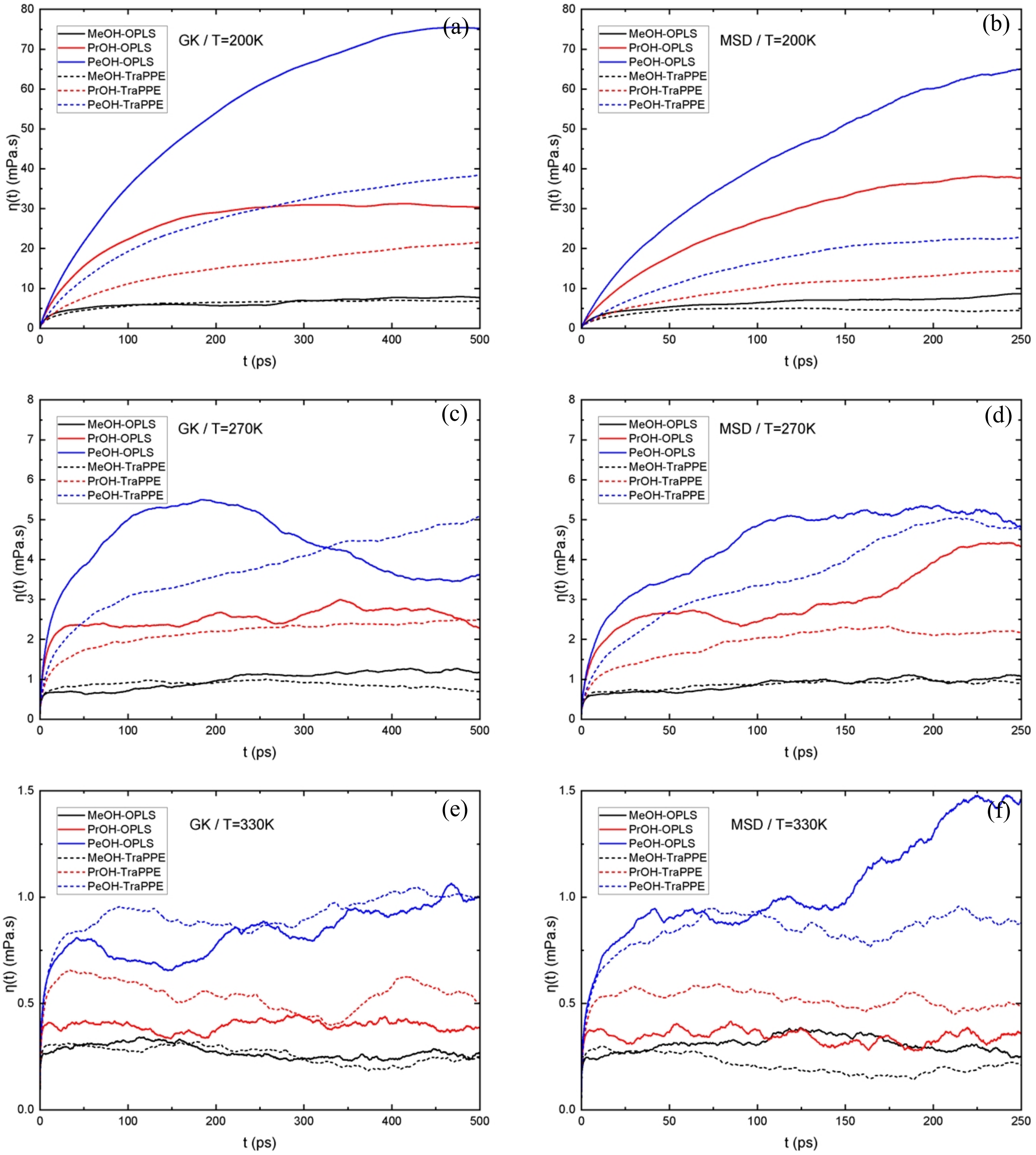

Figure S9: Independents trials for time dependence integral shear viscosity for methanol and 1-hexanol at 240 and 290 K by GK and MSD methods and for both TraPPE-UA and OPLS-AA force fields.

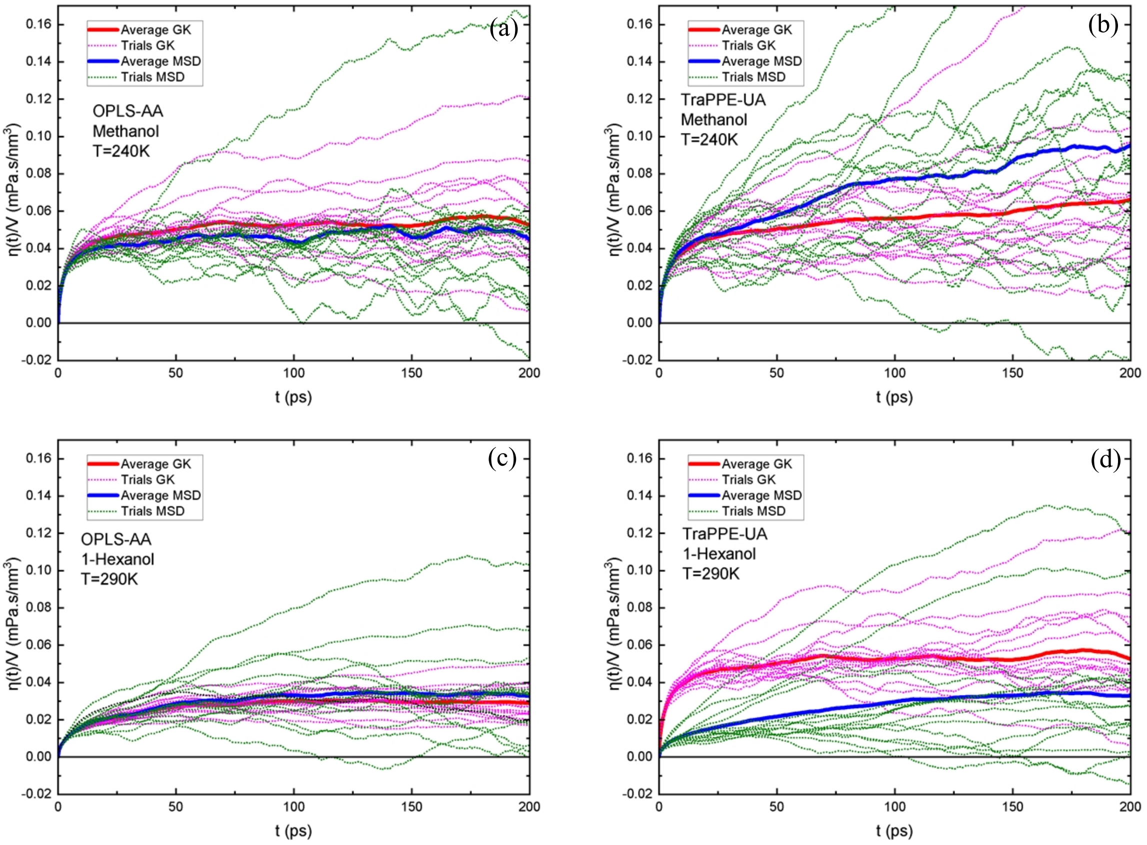

Table S3: Comparison of MD shear viscosity results between this study and some results from other studies.

	T (K)	η (mPa. s)	ref	model	This work (OPLS-AA /GK)	This work (TraPPE-UA /GK)	Exp. (This work Fitt)
methanol	213	3.1	56	Their model	3.503	4.144	2.989
	220	2.0	56	Their model	2.783	3.265	2.471
	240	1.7	56	Their model	1.552	1.783	1.525
	260	1.0	56	Their model	0.947	1.069	1.013
	278.15	0.64	56	Their model	0.643	0.716	0.736
	288	0.62	56	Their model	0.532	0.588	0.629
	298.15	0.57	56	Their model	0.443	0.487	0.541
	308.15	0.47	56	Their model	0.375	0.409	0.471
	318.15	0.41	56	Their model	0.320	0.348	0.414
	328.15	0.42	56	Their model	0.276	0.299	0.366
	340.15	0.29	56	Their model	0.234	0.251	0.319
ethanol	239	3.1	56	Their model	3.571	2.830	4.160
	253.15	2.4	56	Their model	2.358	1.953	2.833
	263	2.2	56	Their model	1.813	1.544	2.221
	273.15	1.7	56	Their model	1.411	1.234	1.761
	280	1.4	56	Their model	1.204	1.071	1.520
	298.15	1.1	56	Their model	0.819	0.758	1.063
	308.15	1.0	56	Their model	0.675	0.638	0.889
	320	0.8	56	Their model	0.545	0.527	0.730
	328.15	0.61	56	Their model	0.475	0.466	0.643
	333	0.60	56	Their model	0.439	0.434	0.597
	338.15	0.47	56	Their model	0.405	0.404	0.554
	343	0.56	58	GAFF	0.376	0.378	0.517
	323	0.82	58	GAFF	0.518	0.503	0.696
	303	1.17	58	GAFF	0.744	0.696	0.974
	298	1.32	58	GAFF	0.821	0.760	1.066
	283	1.93	58	GAFF	1.126	1.008	1.428
1-propanol	263.15	4.72	57	OPLS-AA	2.879	2.147	5.167
	273.15	3.57	57	OPLS-AA	2.123	1.651	3.805
	283.15	2.64	57	OPLS-AA	1.600	1.293	2.863
	293.15	2.03	57	OPLS-AA	1.229	1.030	2.197
	303.15	1.67	57	OPLS-AA	0.961	0.833	1.715
	313.15	1.49	57	OPLS-AA	0.763	0.682	1.360
	323.15	1.20	57	OPLS-AA	0.615	0.566	1.095
	333.15	0.95	57	OPLS-AA	0.502	0.475	0.892
	298.15	1.86	57	OPLS-AA	1.085	0.924	1.937
1-butanol	298.15	2.52	57	OPLS-AA	1.409	1.296	2.537
1-pentanol	298.15	3.22	57	OPLS-AA	2.008	1.590	3.418
1-hexanol	298.15	4.38	57	OPLS-AA	2.887	2.068	4.500

Table S4: Comparison of MD self-diffusion results between this study and some results from other studies.

	T (K)	*D* (10^{-9} $m^2.s^{-1}$)	Ref.	Model	This work (OPLS-AA /MSD)	This work (TraPPE-UA /MSD)	Exp. (This work Fitt)
methanol	213	0.318	56	Their model	0.264	0.164	0.292
	220	0.433	56	Their model	0.346	0.218	0.369
	240	0.750	56	Their model	0.689	0.448	0.669
	260	1.21	56	Their model	1.234	0.826	1.105
	278.15	1.72	56	Their model	1.948	1.333	1.638
	288	2.09	56	Their model	2.436	1.684	1.986
	298.15	2.41	56	Their model	3.019	2.110	2.391
	308.15	2.97	56	Their model	3.680	2.597	2.836
	318.15	3.54	56	Their model	4.430	3.154	3.328
	328.15	4.15	56	Their model	5.273	3.786	3.867
	340.15	4.91	56	Their model	6.411	4.647	4.577
	292	2.36	59	OPLS-AA	2.656	1.844	2.140
	213	0.39	60	OPLS-AA	0.264	0.164	0.292
	240	0.78	60	OPLS-AA	0.689	0.448	0.669
	292	2.16	60	OPLS-AA	2.656	1.844	2.140
	298	2.41	61	Modified OPLS-AA	3.010	2.103	2.385
	308	2.94	61	Modified OPLS-AA	3.670	2.589	2.829
	318	3.46	61	Modified OPLS-AA	4.418	3.145	3.320
	328	4.18	61	Modified OPLS-AA	5.260	3.776	3.859
	338	4.94	61	Modified OPLS-AA	6.197	4.484	4.445
	298	3.12	61	OPLS-AA	3.010	2.103	2.385
	308	3.87	61	OPLS-AA	3.670	2.589	2.829
	318	4.56	61	OPLS-AA	4.418	3.145	3.320
	328	5.43	61	OPLS-AA	5.260	3.776	3.859
	338	6.30	61	OPLS-AA	6.197	4.484	4.445
ethanol	239	0.192	56	Their model	0.166	0.218	0.182
	253.15	0.250	56	Their model	0.292	0.356	0.303
	263	0.363	56	Their model	0.419	0.486	0.418
	273.15	0.493	56	Their model	0.590	0.654	0.568
	280	0.640	56	Their model	0.733	0.790	0.690
	298.15	1.070	56	Their model	1.243	1.247	1.106
	308.15	1.330	56	Their model	1.618	1.567	1.400
	320	1.680	56	Their model	2.167	2.017	1.817
	328.15	2.010	56	Their model	2.616	2.374	2.151
	333	2.200	56	Their model	2.913	2.606	2.368
	338.15	2.400	56	Their model	3.255	2.869	2.616
	290	0.900	59	OPLS-AA	0.989	1.023	0.901
	333	2.400	59	OPLS-AA	2.913	2.606	2.368
	218	0.139	60	OPLS-AA	0.062	0.093	0.076
	239	0.260	60	OPLS-AA	0.166	0.218	0.182
	290	1.030	60	OPLS-AA	0.989	1.023	0.901
	333	2.340	60	OPLS-AA	2.913	2.606	2.368

	298	1.160	61	Modified OPLS-AA	1.238	1.242	1.102
	308	1.526	61	Modified OPLS-AA	1.612	1.562	1.395
	318	1.858	61	Modified OPLS-AA	2.066	1.935	1.742
	328	2.394	61	Modified OPLS-AA	2.607	2.367	2.144
	338	2.870	61	Modified OPLS-AA	3.245	2.861	2.608
	298	1.380	61	OPLS-AA	1.238	1.242	1.102
	308	1.707	61	OPLS-AA	1.612	1.562	1.395
	318	2.216	61	OPLS-AA	2.066	1.935	1.742
	328	2.717	61	OPLS-AA	2.607	2.367	2.144
	338	3.254	61	OPLS-AA	3.245	2.861	2.608
1-propanol	298	0.617	61	Modified OPLS-AA	0.778	0.754	0.605
	308	0.814	61	Modified OPLS-AA	1.056	0.975	0.807
	318	1.101	61	Modified OPLS-AA	1.406	1.240	1.057
	328	1.420	61	Modified OPLS-AA	1.839	1.555	1.362
	338	1.778	61	Modified OPLS-AA	2.368	1.924	1.730
	298	0.898	61	OPLS-AA	0.778	0.754	0.605
	308	1.195	61	OPLS-AA	1.056	0.975	0.807
	318	1.490	61	OPLS-AA	1.406	1.240	1.057
	328	1.954	61	OPLS-AA	1.839	1.555	1.362
	338	2.434	61	OPLS-AA	2.368	1.924	1.730
1-butanol	293	0.403	61	Modified OPLS-AA	0.394	0.449	0.369
	298	0.452	61	Modified OPLS-AA	0.472	0.515	0.433
	303	0.531	61	Modified OPLS-AA	0.564	0.590	0.504
	293	0.474	61	OPLS-AA	0.394	0.449	0.369
	298	0.555	61	OPLS-AA	0.472	0.515	0.433
	303	0.632	61	OPLS-AA	0.564	0.590	0.504
1-pentanol	298	0.301	61	Modified OPLS-AA	0.280	0.398	0.282
	298	0.360	61	OPLS-AA	0.280	0.398	0.282
1-hexanol	298	0.218	61	Modified OPLS-AA	0.188	0.274	0.203
	298	0.222	61	OPLS-AA	0.188	0.274	0.203

Figure S10: Temperature dependence of the predicted Stoke-Einstein effective hydrodynamic radius from MD simulations for pure 1-alkanol liquids by using GK-average (GK+MSD) and MSD-average (GK+MSD) methods for self-diffusion and shear viscosity respectively for OPLS-AA and TraPPE-UA force fields.

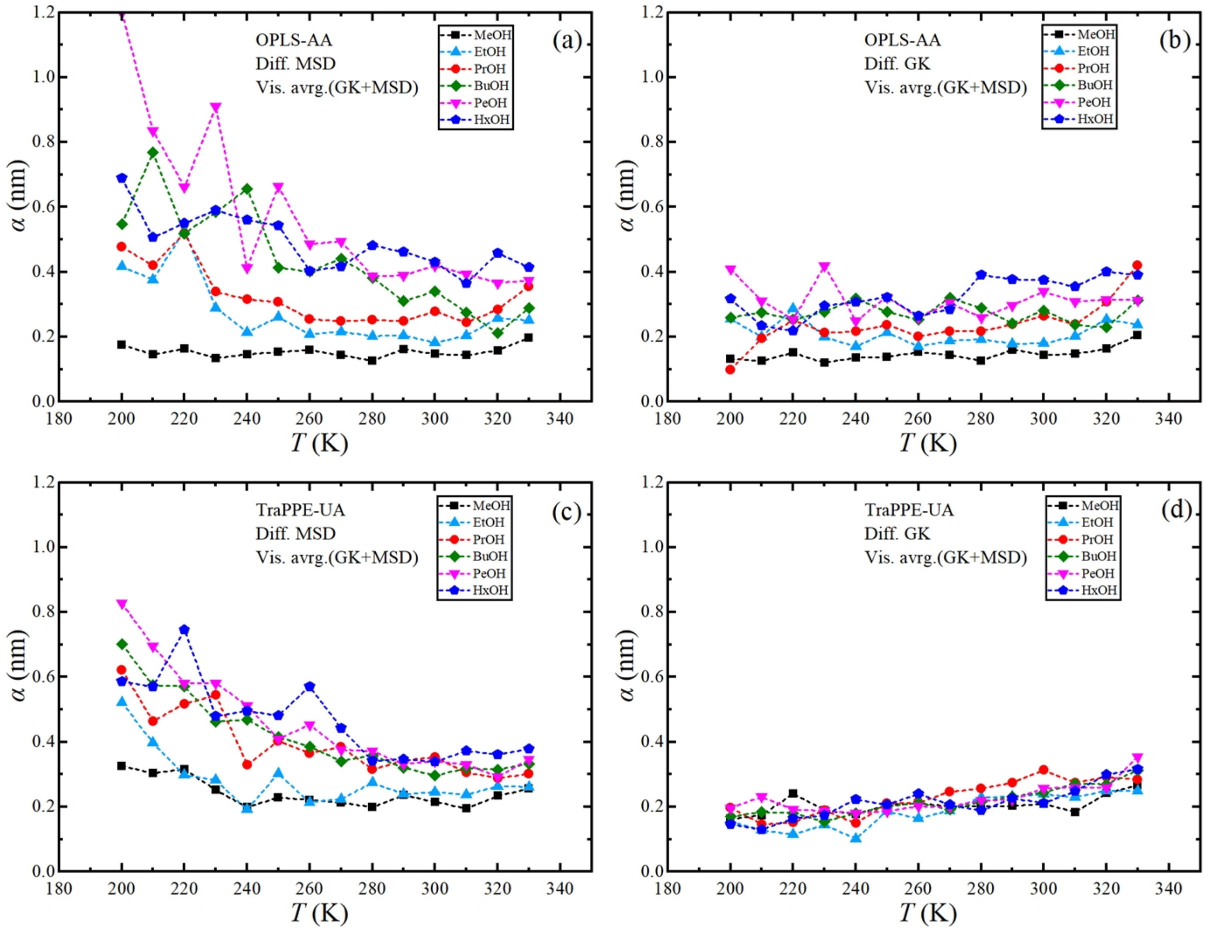

Supplement: RA-014-D4RA03494E-s001 [file RA-014-D4RA03494E-s001.pdf]
